# Hypoxia-induced macropinocytosis represents a metabolic route for liver cancer

**DOI:** 10.1038/s41467-022-28618-9

**Published:** 2022-02-17

**Authors:** Misty Shuo Zhang, Jane Di Cui, Derek Lee, Vincent Wai-Hin Yuen, David Kung-Chun Chiu, Chi Ching Goh, Jacinth Wing-Sum Cheu, Aki Pui-Wah Tse, Macus Hao-Ran Bao, Bowie Po Yee Wong, Carrie Yiling Chen, Chun-Ming Wong, Irene Oi-Lin Ng, Carmen Chak-Lui Wong

**Affiliations:** 1 Department of Pathology, Li Ka Shing Faculty of Medicine, The University of Hong Kong, Hong Kong, China; 2 State Key Laboratory of Liver Research, The University of Hong Kong, Hong Kong, China

**Keywords:** Cancer metabolism, Hepatocellular carcinoma

## Abstract

Hepatocellular carcinoma (HCC) invariably exhibits inadequate O_2_ (hypoxia) and nutrient supply. Hypoxia-inducible factor (HIF) mediates cascades of molecular events that enable cancer cells to adapt and propagate. Macropinocytosis is an endocytic process initiated by membrane ruffling, causing the engulfment of extracellular fluids (proteins), protein digestion and subsequent incorporation into the biomass. We show that macropinocytosis occurs universally in HCC under hypoxia. HIF-1 activates the transcription of a membrane ruffling protein, EH domain-containing protein 2 (EHD2), to initiate macropinocytosis. Knockout of HIF-1 or EHD2 represses hypoxia-induced macropinocytosis and prevents hypoxic HCC cells from scavenging protein that support cell growth. Germline or somatic deletion of *Ehd2* suppresses macropinocytosis and HCC development in mice. Intriguingly, EHD2 is overexpressed in HCC. Consistently, HIF-1 or macropinocytosis inhibitor suppresses macropinocytosis and HCC development. Thus, we show that hypoxia induces macropinocytosis through the HIF/EHD2 pathway in HCC cells, harnessing extracellular protein as a nutrient to survive.

## Abstract (web summary)

Cancer cells rely on macropinocytosis to scavenge extracellular proteins for growth. Here the authors show that macropinocytosis supports the survival of hypoxic hepatocellular carcinoma cells and this is dependent on HIF-1, which in turns activates the transcription of a membrane ruffling protein, EH domain-containing protein 2.

## Introduction

Hepatocellular carcinoma (HCC), a malignancy that arises from hepatocytes, accounts for 90% of primary liver cancer cases. HCC ranks as the second most lethal cancer worldwide. As HCC is asymptomatic, most patients are not amenable to surgical treatment at diagnosis. For advanced HCC, two tyrosine-kinase inhibitors (TKIs), sorafenib and lenvatinib, are the major first-line targeted therapies, but they have a modest survival benefit of only 3 months^1,2^. Another TKI, regorafenib, and an immune-checkpoint inhibitor, nivolumab (anti-PD-1 antibody), are the second-line treatments for sorafenib-resistant HCC^3,4^. Excitingly, combined treatment of bevacizumab (anti-VEGF antibody) and atezolizumab (anti-PD-L1 antibody) has been recently recruited as a first-line treatment in unresectable HCC patients with improved survival benefits than sorafenib^5^. Despite the increasing therapeutic options, the median life expectancy of HCC patients is still shorter than 2 years. Efforts should be continued to reveal new molecular cascades supporting HCC cell survival for the identification of more effective therapeutic targets.

Hypoxia, a decline in the oxygen (O_2_) content, is universally found in all solid cancers due to the abnormal vasculature. Cells adapt to hypoxia through hypoxia-inducible factors (HIFs), which are heterodimeric proteins consisting of the HIF-1/-2α and HIF-1β subunits^6^. In an O_2_-rich environment, prolyl hydroxylase (PHD) uses O_2_ to hydroxylate the proline resides of HIF-1/2α, thereby allowing the binding of von Hippel–Lindau (VHL), an E3 ubiquitin ligase^7^. VHL drives the ubiquitin-mediated proteasomal degradation of HIF-1/-2α^8^. In an O_2_-deficient environment, HIF-1/2α is stabilized and translocates into the nucleus, where it binds to the constitutively expressed HIF-1β subunit and, together with coactivators, recognizes genomic sequences encompassing hypoxia-response elements (HREs) to turn on the transcriptional machinery of genes^9^. HIFs are frequently detected in solid cancers and correlated with poor clinical outcomes^10^. HIFs are particularly important in the metabolic adaptation of hypoxic cancer cells. HIFs rechannel metabolites into glycolysis instead of oxidative phosphorylation (OXPHOS). Although glycolysis produces less energy than OXPHOS, HIFs regulate multiple genes to accelerate glycolytic flux to compensate. HIFs induce the expression of glucose transporter type 1 (GLUT1) to increase glucose uptake, that of almost all glycolytic enzymes^11^, and pyruvate dehydrogenase kinase 1 (PDK1) to impede metabolic entry into the TCA cycle^12^, and that of lactate-dehydrogenase A (LDHA) to convert pyruvate into lactate^13^. HIFs also induce the less-active electron-transport-chain components, NUDFA4 mitochondrial complex-associated-like 2 (NDUFA4L2) and cytochrome c oxidase subunit IV isoform 2 (COX4–2), to decelerate electron transfer and reduce ROS accumulation^14,15^.

Cancer growth relies on glucose, glutamine (Gln), and amino acids (AAs). When these nutrients become limited, cancer cells rely on macropinocytosis to scavenge extracellular nutrients to use as fuels^16^. Macropinocytosis initiates from actin-dependent ruffling and invagination of the plasma membrane. The ruffled membrane folds back to form a closed macropinocytic cup containing extracellular fluids^16^. The macropinocytic cup detaches from the membrane as a vesicle, named the macropinosome, subsequently enters the cytoplasm and later fuses with lysosomes^17^. Lysosomes digest the components within the macropinosomes and release biomass that can be harnessed to support cancer growth. Albumin, the most abundant protein in the human body, is synthesized in the liver and transported by the blood, acting as a carrier for molecules with low water solubility and a rheostat to maintain intravascular pressure^18^. However, albumin frequently accumulates in tumors as a result of abnormal tumor lymphatics and blood vessels^18^. Reusing the proteins trapped within tumors creates an advantage for cancer growth by bypassing *de novo* anabolism, which is especially important when tumors are experiencing limited nutrients^19^. Apart from scavenging extracellular proteins, macropinocytosis has also been shown to be used by cancer cells to take up necrotic cell debris and reuse the proteins, lipids, and nucleotides from dying cells as a nutrient source when the nutrient supply is inadequate in recent studies^20,21^.

Membrane ruffling is the first and critical step in macropinocytosis, which greatly depends on actin-cytoskeleton rearrangement to support the invagination of the plasma membrane. Multiple studies have provided converging evidence showing that the Rac-family small GTPase 1 (RAC1), a RhoGTPase that controls actin remodeling and cytoskeleton rearrangement, is essential to macropinocytosis by supporting membrane ruffle formation^21^. Macropinocytosis in PTEN-deficient cancer cells is dependent on AMPK, which in turn activates RAC1 to initiate macropinocytosis^21^. Recently, an elegant study showed that RAS promotes the translocation of vacuolar ATPase (V-ATPase) to the plasma membrane, which facilitates RAC1 to induce membrane ruffle formation^22^.

Here, we show that macropinocytosis is universally induced by hypoxia in all HCC cell lines tested. Hypoxia is frequently observed in solid cancers, including HCC. As hypoxic regions are poorly perfused and have limited nutrients, we posit that hypoxic cancer cells should have a greater reliance on extracellular proteins than normoxic cancer cells. Albumin is mainly produced by hepatocytes. We postulate that the albumin produced by neighboring hepatocytes could be harnessed by HCC cells as a nutrient source. We observe that hypoxia-induced macropinocytosis is abated when HIF-1α is knocked down/out or inhibited. Interestingly, we identify a HIF transcriptional target, EH-domain-containing protein 2 (EHD2), that drives membrane ruffle formation and macropinocytosis in hypoxic HCC cells. Germline or somatic deletion of *Ehd2* in mice prevents the development of HCC induced by hepatocarcinogen or liver-specific *Tp53* knockout (KO) or *Keap1* KO with *c-Myc* overexpression (OE). HIF-1/EHD2 is a regulatory pathway in macropinocytosis. The inhibition of macropinocytosis represents a potent therapeutic approach for HCC.

## Results

### Hypoxia induces macropinocytosis in HCC cells

**Fig. 1:**
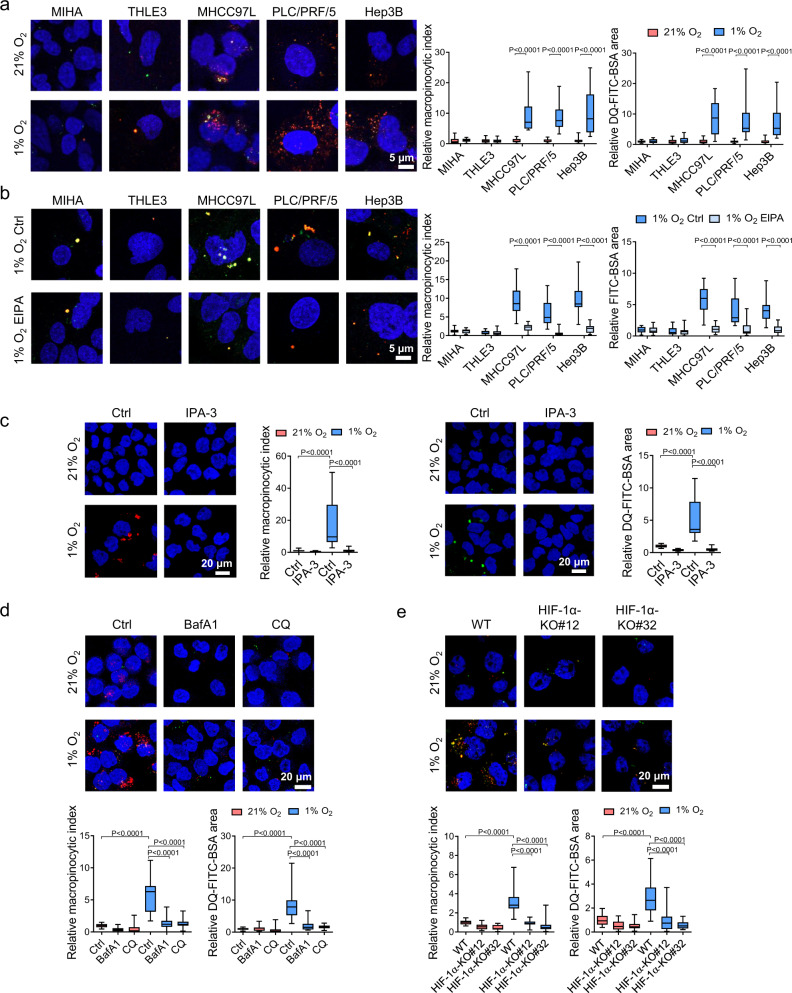
Hypoxia induces macropinocytosis in HCC cells. **a** Immortalized liver cells MIHA, THLE3, and HCC cells MHCC97L, PLC/PRF/5, and Hep3B exposed to 21 and 1% O_2_ were incubated with 70 kDa tetramethylrhodamine dextran (TMR dextran) and dye-quenched FITC-conjugated BSA (DQ-FITC-BSA) for macropinosome labeling and measurement of protein degradation, respectively. Confocal images show dextran uptake/macropinosomes (red), degraded FITC-BSA (green), and nuclei (DAPI, blue). Macropinocytic indexes were calculated based on the intensities of red signals, DQ-FITC-BSA particle areas were calculated from green signals, respectively. Values were normalized to 21% O_2_. **b** MIHA, THLE3, MHCC97L, PLC/PRF/5, and Hep3B exposed to 1% O_2_ were treated with 50 µM EIPA or vehicle control (Ctrl). Confocal images demonstrate the macropinosome (dextran, red) and constitutively fluorescent BSA (green) within the cells. Macropinocytic indexes were calculated based on the intensities of red signals, constitutively fluorescent BSA particle areas were calculated from green signals. Values were normalized to 21% O_2_ Ctrl. **c** Left: MHCC97L cells were treated with 10 µM IPA-3 or vehicle control (Ctrl, DMSO). Confocal images show dextran uptake/macropinosomes (red) and nuclei (DAPI, blue) of MHCC97L cells exposed to 21 and 1% O_2_. Right: Confocal images show degraded FITC-BSA (green) and nuclei (DAPI, blue) of MHCC97L cells exposed to 21 and 1% O_2_. Macropinocytic indexes and DQ-FITC-BSA particle areas were normalized to 21% O_2_ Ctrl. **d** MHCC97L cells exposed to 21 and 1% O_2_ were treated with 200 nM lysosomal inhibitors bafilomycin A1 (BafA1), 20 μM chloroquine (CQ), or vehicle control (Ctrl, DMSO). Confocal images show dextran uptake/macropinosomes (red), degraded FITC-BSA (green), and nuclei (DAPI, blue). Macropinocytic indexes and DQ-FITC-BSA particle areas were normalized to 21% O_2_ Ctrl. **e** Confocal images demonstrate dextran uptake/macropinosomes (red), degraded DQ-FITC-BSA (green), and nuclei (DAPI, blue) of MHCC97L wild type (WT), -HIF-1α-KO#12, and -HIF-1α-KO#32 exposed to 21 and 1% O_2_. Macropinocytic indexes and DQ-FITC-BSA particle areas were normalized to values of 21% O_2_ WT. **a**–**e** Results were from 3 independent experiments. Box-and-whisker: center line, median; box limits, 25th to 75th percentiles; whiskers, min to max. Two-way ANOVA with Bonferroni correction. Source data are provided as a Source Data file.

To study whether hypoxia affects the macropinocytic capacity of HCC cells, we evaluated the ability of HCC cells to form macropinosomes and degrade engulfed proteins under 21 and 1% O_2_ conditions. We incubated human HCC cell lines, MHCC97L, PLC/PRF/5, and Hep3B and human immortalized liver cells, MIHA and THLE3, that were pre-exposed to 21 and 1% O_2_ for 36 h with tetramethylrhodamine-labeled high-molecular-weight dextran (TMR dextran, 70 kDa), a standard and well-established marker of macropinocytosis^23–25^. Simultaneously, we added dye-quenched FITC-conjugated bovine serum albumin (DQ-FITC-BSA) (65 kDa), which only emits fluorescence when BSA undergoes proteolytic degradation through the lysosomal pathway^26^. Strikingly, confocal imaging clearly showed that hypoxia increased dextran uptake and BSA proteolysis in HCC cell lines but not in normal liver cell lines (Fig. 1a). Macropinocytosis (calculated as the macropinocytic index) was consistently induced by hypoxia in all human HCC cell lines we tested. We next asked whether hypoxia-induced macropinocytosis could be blocked by the inhibitors 5-(N-ethyl-N-isopropyl) amiloride (EIPA), IPA-3, bafilomycin A1 (BafA1), and chloroquine (CQ), which target different steps in macropinocytosis. EIPA is a Na^+^/H^+^ exchanger inhibitor that blocks macropinocytosis by lowering the membranous pH, thereby suppressing RAC1 activity^27^. We incubated immortalized normal liver and HCC cell lines with dextran and constitutively fluorescent BSA, which can clearly indicate the internalization of BSA together with dextran. In all, 50 µM EIPA effectively blocked dextran and BSA uptake under hypoxic conditions, but has little effect on normoxic conditions that maintained a low level of macropinocytosis (Fig. 1b and Supplementary Fig. 1a). IPA-3 targets PAK1 to inhibit membrane ruffling and the closure of macropinocytic cups^28^. BafA1 is lysosomal inhibitor and V-ATPase inhibitor, while CQ is a lysosomal inhibitor that blocks the degradation of engulfed proteins^29,30^. IPA-3, BafA1, and CQ all abolished hypoxia-induced dextran uptake and BSA degradation in MHCC97L cells (Fig. 1c, d).

Apart from macropinocytosis, it is reported that albumin can be internalized through other endocytic pathways such as caveolae-dependent endocytosis and clathrin-mediated endocytosis^31,32^. We knocked down caveolin1 (*CAV1*) or clathrin heavy chain (*CLTC*) in MHCC97L cells and evaluated macropinocytic index and BSA proteolysis in the cells (Supplementary Fig. 1b, d). CAV1 is the main component of caveolae, which are flask-shaped invaginations of plasma membrane participated in caveolae-dependent endocytosis^33^. CLTC interacts with clathrin light chain to form a clathrin “triskelion”, that composes of the structural backbone of the clathrin coat to promote membrane bending during endocytosis^34^. After knocking down the key components of caveolae- or clathrin-dependent endocytic pathways, the levels of dextran uptake and BSA degradation remain unchanged (Supplementary Fig. 1c, e). All these data confirmed that hypoxia-induced protein scavenging is independent of the caveolae- and clathrin-mediated endocytic pathways.

HIF-1 is the master regulator that controls almost all adaptive responses to hypoxia. Hypoxia-induced macropinosome formation and BSA degradation were abrogated by HIF-1α-KO (Fig. 1e and Supplementary Fig. 2a) or treatment with the well-established HIF inhibitor digoxin (dig)^35^ in MHCC97L cells (Supplementary Fig. 2b, c). Together, these findings suggest that hypoxia induces macropinocytosis through HIF-1.

### Protein scavenging supports the survival of hypoxic HCC cells

**Fig. 2:**
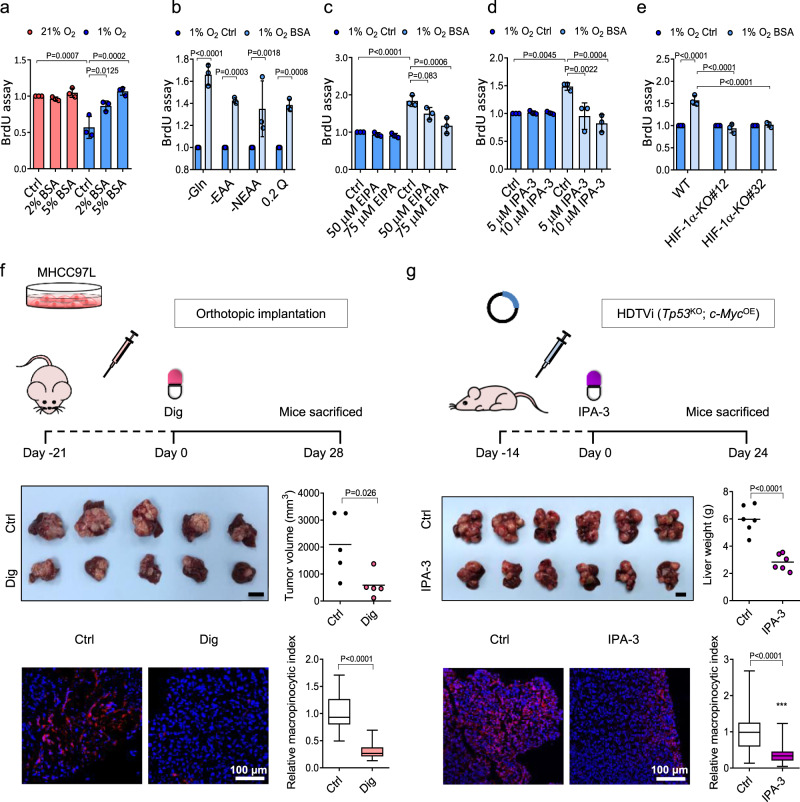
Protein scavenging promotes hypoxic HCC cell growth. **a** Cell proliferation of MHCC97L cells cultured in 0.2 mM glutamine (0.2 Q) supplemented with increasing doses of BSA or vehicle control (Ctrl) in 21 and 1% O_2_. Values were normalized to 21% O_2_ Ctrl. **b** Cell proliferation of MHCC97L cells cultured in glutamine-depleted (-Gln), essential amino acid-depleted (-EAA), nonessential amino acid-depleted (-NEAA), or 0.2 Q DMEM media in 1% O_2_ treated with 5% BSA or vehicle control (Ctrl). **c** Cell proliferation of MHCC97L cells cultured in 10% AA-DMEM containing 5% BSA. Cells were treated with EIPA or vehicle control (Ctrl). **d** Cell proliferation of MHCC97L cells cultured in 10% AA-DMEM treated with IPA-3 or vehicle control (Ctrl). Cells were supplemented with 5% BSA or vehicle control (Ctrl). **e** Cell proliferation of MHCC97L wild type (WT), -HIF-1α-KO#12, and -HIF-1α-KO#32 cultured in 10% AA-DMEM treated with 5% BSA or vehicle control (Ctrl, PBS) under 1% O_2_ condition. **f** Top: 6–8 week-old male BALB/cAnN-nude mice were orthotopically implanted with luciferase-labeled MHCC97L cells into the livers and received digoxin (dig) or vehicle-control (Ctrl) treatment for 28 days. Middle: Image of orthotopic HCC xenografts and tumor volumes of mice. Bottom: Confocal images show dextran uptake/macropinosomes (red) and nuclei (DAPI, blue) in HCC tissues. *n* = 5 biologically independent samples. **g** Top: Hydrodynamic tail-vein injection (HDTVi) of genome-editing systems was performed in 8–10-week-old male C57BL/6 N mice to induce *Tp53*^KO^; *c-Myc*^OE^ mouse HCC. Mice received IPA-3 or vehicle control (Ctrl) treatment for 24 days. Middle: Image of HCC and liver weights of mice. Bottom: Confocal images show dextran uptake/macropinosomes (red) and nuclei (DAPI, blue) of HCC tissues. *n* = 6 biologically independent samples. **a**–**e** Results were from 3 independent experiments. **b**–**e** Values were normalized to 1% O_2_ Ctrl. **f**, **g** Scale bars, 1 cm. Macropinocytic indexes were normalized to Ctrl. Error bars indicate mean ± SD. Scatter plot: center line, mean. Box-and-whisker: center line, median; box limits, 25th to 75th percentiles; whiskers, min–max. **a**–**e** Two-way ANOVA with Bonferroni correction. **f**, **g** Two-tailed Student’s *t*-test. Source data are provided as a Source Data file.

Next, we sought to explore whether macropinocytosis facilitates the survival of hypoxic HCC cells. To address whether extracellular proteins support hypoxic HCC cell growth, we employed BrdU assay and XTT assay to measure the cell proliferation of MHCC97L cells cultured in low-glutamine DMEM (0.2 mM glutamine, 0.2 Q). XTT is a metabolic-based cell proliferation assay, while BrdU is a synthetic thymidine analog that incorporates into DNA during cell division. Both assays demonstrated that exogenous BSA increased cell proliferation in a dose-dependent manner in HCC cells exposed to 1% O_2_ but not those exposed to 21% O_2_ (Fig. 2a and Supplementary Fig. 3a). Exogenous BSA also consistently restored the growth of hypoxic HCC cells in different nutrient-deficient conditions, including glutamine depletion (-Gln), essential amino acid depletion (-EAA), nonessential amino acid depletion (-NEAA), or 0.2 Q conditions (Fig. 2b and Supplementary Fig. 3b). Treatment of EIPA or IPA-3 abolished the growth-promoting effects of exogenous BSA in a dose-dependent manner (Fig. 2c, d and Supplementary Fig. 3c). Notably, BSA failed to rescue cell growth in 1% O_2_ when HIF-1α was knocked out (Fig. 2e and Supplementary Fig. 3d), suggesting an important role for HIF-1 in protein scavenging-dependent cell survival in hypoxia. The rescuing effect of BSA treatment was less significant in normoxic conditions (Supplementary Fig. 3e–h). Hypoxia not only reduces cell proliferation but induces apoptosis^36^. We measured the percentage of dead cells in different nutrient-deficient conditions with or without BSA treatment exposed to 21 and 1% O_2_. SYTOX Green signal, which indicates cell death, was increased under hypoxic conditions (Supplementary Fig. 3i–l). However, the treatment of BSA did not have significant effect on cell death, indicating that the scavenged proteins are promoting cell growth but not affecting cell death (Supplementary Fig. 3i–l).

Pharmacological inhibition of HIF-1 using digoxin suppressed the growth of orthotopic tumors derived from MHCC97L cells (Fig. 2f) and lung metastasis in vivo (Supplementary Fig. 4). Macropinocytic capacity of tumors was measured by ex vivo dextran labeling of tumor tissues^37^. Ex vivo analysis showed that the macropinocytic index in HCC tumors from mice administered with digoxin was significantly reduced as compared with control tumors (Fig. 2f).

Consistently, IPA-3 blocked the growth of MHCC97L-derived subcutaneous xenografts and macropinocytosis without affecting mouse body weight (Supplementary Fig. 5). To ensure that the effect is not limited to tumors derived from MHCC97L cells, we induced HCC by hydrodynamic tail-vein injection (HDTVi) of genome-editing systems, including the CRISPR–Cas9 and Sleeping Beauty transposon systems, which enabled the deletion of *Tp53* and OE of *c-Myc* in the liver of immunocompetent mice and formation of *Tp53*^KO^; *c-Myc*^OE^ HCC tumors^38^. Strikingly, IPA-3 profoundly suppressed the growth and macropinocytosis of *Tp53*^KO^; *c-Myc*^OE^ HCC tumors (Fig. 2g) without affecting mouse body weight (Supplementary Fig. 6). We showed that exogenous proteins benefit the growth of HCC, and that the growth-promoting effect could be abolished by a HIF-1 inhibitor or IPA-3.

### HIF-1 induces macropinocytosis through transcriptional activation of EHD2

**Fig. 3:**
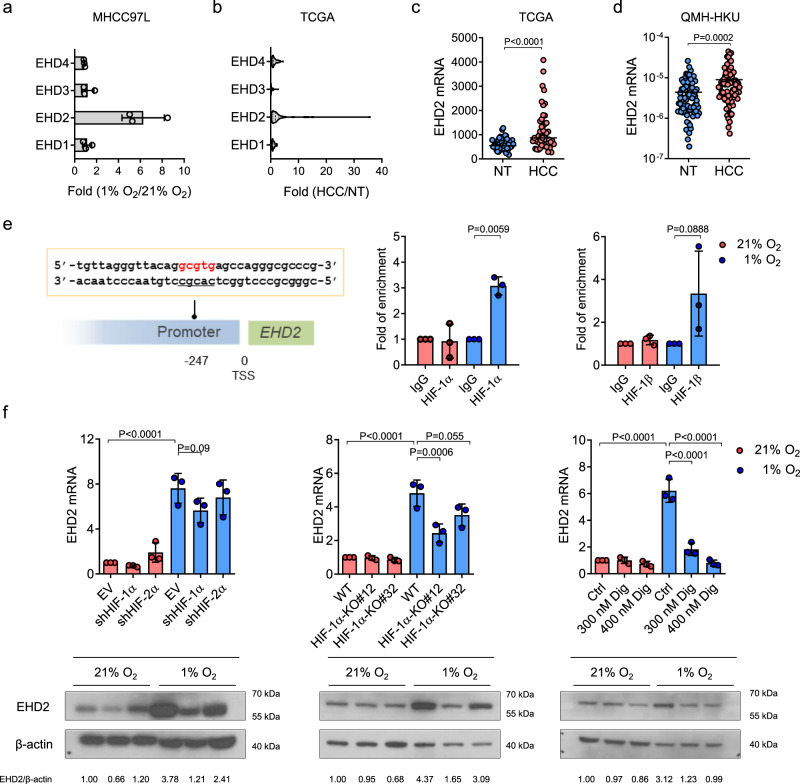
EHD2 is a HIF transcriptional target overexpressed in HCC. **a** RT-qPCR data showed mRNA expressions of EHD-family members (EHD1–EHD4) in MHCC97L cells exposed to 21 and 1% O_2_. Values were normalized to 21% O_2_. mRNA expressions were normalized to 18S. The results were from 3 independent experiments. **b** Transcriptome sequencing data from the TCGA show the mRNA expressions of EHD1–EHD4 in 49 pairs of human HCC tissues and nontumorous liver tissues (NT). *n* = 49 biologically independent samples. **c** EHD2 mRNA expressions in HCC and corresponding NT tissues from the TCGA. **d** EHD2 mRNA expressions in HCC and corresponding NT tissues from patients admitted to Queen Mary Hospital, the University of Hong Kong (QMH-HKU). **e** Left: A putative hypoxia-response element (HRE) in *EHD2*. Transcription start site (TSS) is defined as 0. Right: ChIP assay was performed in MHCC97L cells exposed to 21 and 1% O_2_ using IgG, HIF-1α, or HIF-1β antibodies. The fold of enrichment was referenced to IgG. **f** EHD2 mRNA and protein expressions in MHCC97L empty vector (EV), -shHIF-1α, -shHIF-2α cells (left), MHCC97L-wild type (WT), -HIF-1α-KO#12, and -HIF-1α-KO#32 cells (middle), and MHCC97L treated with 300 nM digoxin (Dig), 400 nM Dig, or vehicle ctrl (Ctrl) (right) exposed to 21 and 1% O_2_. mRNA expressions were normalized to 18S. Band intensities were normalized to 21% MHCC97L EV, -WT, or -Ctrl. **e**–**f** (upper panel): Results were from 3 independent experiments. **f** (lower panel): Results were representative for 3 independent experiments with similar results from different repeats. Error bars indicate mean ± SD. Two-way ANOVA with Bonferroni correction. Source data are provided as a Source Data file.

We next investigated the molecular mechanism by which HIF-1 activates macropinocytosis. Macropinocytosis is initiated by membrane ruffling, which relies on actin remodeling^28^. Members of the Eps15 homology-domain (EHD) family are closely linked with actin reorganization through direct and indirect binding with actin filaments^39–41^. The EHD family consists of 4 members in humans, EHD1–EHD4. All EHDs are dynamin-related ATPases that interact with the lipids in the plasma membrane and maintain the plasma membrane curvature to induce membrane fission for the formation of vesicles to control different endocytic processes^42^. The structure of EHDs is highly homologous, containing an ATP-binding domain, a linker region, and an Eps15 homology (EH) domain^42^. ATP binding is crucial to EHD plasma membrane attachment. After ATP is hydrolyzed, EHDs detach from the membrane, causing membrane fission and vesicle formation^42^. To study whether EHDs contribute to hypoxia-induced macropinocytosis in HCC cells, we evaluated the expression levels of EHDs in various HCC cell lines exposed to 21% or 1% O_2_. Interestingly, RT-qPCR results showed that EHD2 was induced by hypoxia in all HCC cells (Fig. 3a and Supplementary Fig. 7a). A dataset from the TCGA database comprising transcriptome-sequencing data from 49 pairs of human HCC and nontumorous (NT) tissues showed that EHD2 was the only member overexpressed (Fig. 3b, c). Data from the TCGA database also showed that EHD2 was overexpressed in other cancer types such as esophageal carcinoma (ESCA), kidney renal clear-cell carcinoma (KIRC), and intrahepatic cholangiocarcinoma (ICC) (Supplementary Fig. 7b). RT-qPCR analysis of an independent cohort of HCC patients admitted to our hospital (Queen Mary Hospital, the University of Hong Kong, QMH-HKU) confirmed that EHD2 was significantly overexpressed in HCC tissues (Fig. 3d). Waterfall plots showed that 38.8% and 48.8% of HCC patients showed at least 2-fold upregulation of EHD2 mRNA expression in HCC tissues relative to their NT counterparts in the TCGA database and QMH-HKU cohort, respectively (Supplementary Fig. 7c). Clinicopathological analysis further demonstrated that EHD2 overexpression was significantly correlated with a large tumor size in human HCC patients (Table 1).

**Table 1:** Clinicopathological correlation of EHD2 overexpression in human HCC.

Clinicopathological phenotype	Number of cases (%)	Mean	*P*-value
Tumor size			
• ≤5 cm	29	0.2283	0.017*
• >5 cm	44	1.1430	
Direct liver invasion			
• Absent	39	1.0936	0.052
• Present	29	0.2941	
Sex			
• Male	50	1.1520	0.099
• Female	16	0.0469	
Chronic liver disease			
• Normal	11	0.1827	0.202
• Chronic liver disease and cirrhosis	63	0.8594	
Cellular differentiation by Edmondson grading			
• I–III	37	0.5505	0.253
• IV–VI	36	0.9875	
Tumor microsatellite formation			
• Absent	33	0.6415	0.482
• Present	41	0.9117	
Venous invasion			
• Absent	26	1.095	0.493
• Present	40	0.7905	
Cirrhotic liver			
• Normal and chronic hepatitis	31	0.8974	0.534
• Cirrhosis	43	0.6588	
pTNM stage			
• I–II	26	0.6450	0.632
• I–IV	48	0.8360	
Hepatitis C surface antigen by IHC			
• Absent	22	0.6800	0.676
• Present	12	0.9100	
Hepatitis B surface antigen by IHC			
• Absent	18	0.6756	0.858
• Present	52	0.7762	
Plasma hepatitis B surface antigen			
• Absent	14	0.8950	0.983
• Present	53	0.8808	
Tumor encapsulation			
• Absent	44	0.8225	0.94
• Present	25	0.8552	

Mean: average values of EHD2 expression in human HCC samples relative to their adjacent non-tumorous (NT) tissues determined using the formula: ΔCt^HCC(EHD2-18S)^ - ΔCt^NT(EHD2-18S)^.

pTNM: pathological tumor-node-metastasis. Two tailed *t*-test: *P < 0.05.

Next, we asked if EHD2 is a direct transcriptional target of HIF-1. A putative HRE (-A/GCGTG) was found in the promoter of *EHD2*. Chromatin immunoprecipitation (ChIP) assay confirmed the binding of HIF-1α and HIF-1β to the HRE of *EHD2* (Fig. 3e). RT-qPCR and western blotting confirmed that hypoxia-induced EHD2 mRNA and protein expression in MHCC97L cells was abolished upon knockdown (KD) or KO of HIF-1α and slightly attenuated upon KD of HIF-2α (Fig. 3f and Supplementary Fig. 8). Digoxin also abrogated hypoxia-induced EHD2 expression (Fig. 3f). Our data confirm that EHD2, a gene important for the actin remodeling involved in membrane ruffling, is a direct transcriptional target of HIFs.

### EHD2 promotes hypoxia-induced macropinocytosis in HCC cells

**Fig. 4:**
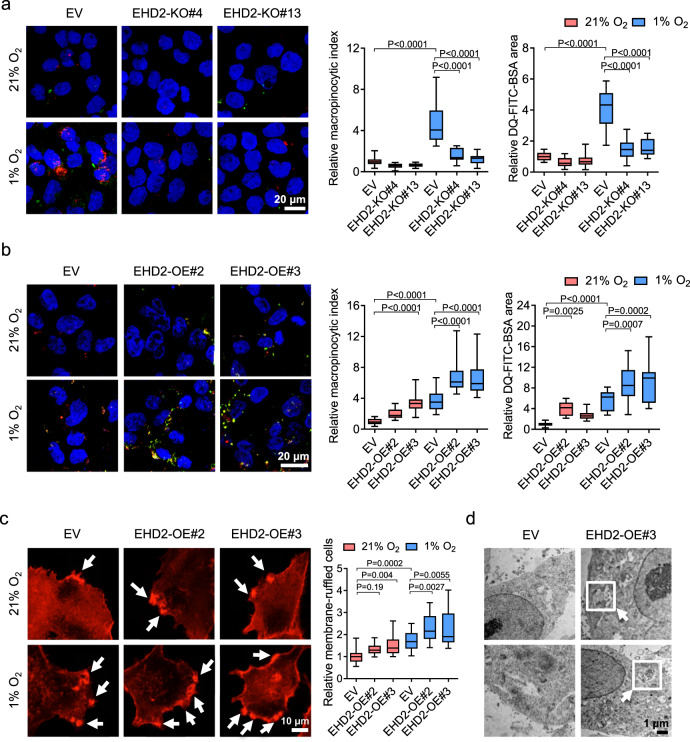
EHD2 promotes macropinocytosis through actin remodeling. **a**. Confocal images show dextran uptake/macropinosomes (red) and FITC-BSA degradation (green) of MHCC97L-EV, -EHD2-KO#4, and -EHD2-KO#13 exposed to 21 and 1% O_2_. **b** Confocal images reveal dextran uptake/macropinosomes (red) and FITC-BSA degradation (green) of MHCC97L-EV, -EHD2-OE#2, and -EHD2-OE#3 cells exposed to 21 and 1% O_2_. **c** Super high-resolution images show actin filaments (phalloidin, red) in MHCC97L-EV, -EHD2-OE#2, and -EHD2-OE#3 cells exposed to 21 and 1% O_2_. Arrows indicate the membrane ruffles. Percentage of cells with membrane ruffles was normalized to 21% O_2_ EV. **d** Images acquired by transmission electronic microscope (TEM) in MHCC97L-EV and -EHD2-OE#3 cells exposed to 21% O_2_. Arrows point to the macropinosomes in MHCC97L-EHD2-OE cells. **a**, **b** Macropinocytic indexes and DQ-FITC-BSA particle areas were normalized to 21% O_2_ EV. **a**–**c** Results were from 3 independent experiments. **d** Results were representative from *n* = 5 imaging analysis. Scatter plot: center line, mean. Box-and-whisker: center line, median; box limits, 25th to 75th percentiles; whiskers, min to max. Two-way ANOVA with Bonferroni correction. Source data are provided as a Source Data file.

EHD2 has been shown to bind to multiple proteins to promote endocytosis. The N-terminus of EHD2 binds to the cell membrane, while the C-terminus of EHD2 binds to the actin-binding protein EH-domain-binding protein 1 (EHBP1) to mediate cytoskeleton rearrangement, driving membrane curvature to facilitate endocytosis^41^. EHD2 also interacts with NEK3, which binds to a Rho-family guanine exchange factor (GEF) to activate Rac1 to support actin-dependent endocytosis^39^. The roles of EHD2 in endocytosis prompted us to speculate that EHD2 may be responsible for hypoxia/HIF-induced macropinocytosis, which also relies on actin-dependent membrane ruffle formation. We established EHD2-KO or EHD2-KD subclones of MHCC97L and PLC/PRF/5 cells and evaluated their macropinocytic capacities in 21 and 1% O_2_ conditions (Fig. 4a and Supplementary Fig. 9a, c). KO and KD of EHD2 remarkably obliterated the hypoxia-induced effects on dextran uptake and BSA degradation (Fig. 4a and Supplementary Fig. 9c). We further generated EHD2-OE in MHCC97L and Hep3B cells with a CRISPR–dCas9 Synergistic Activation Mediator-activating system (Fig. 4b and Supplementary Fig. 9b, d). EHD2-OE subclones exhibit enhanced capabilities to take up dextran and degrade BSA, even in 21% O_2_ (Fig. 4b and Supplementary Fig. 9d). OE of EHD2 further enhanced dextran uptake and BSA degradation in 1% O_2_ (Fig. 4b).

We further employed PHD inhibitor dimethyloxallyl glycine (DMOG) and VHL inhibitor VH-298, which both induce HIF. DMOG is an α-ketoglutarate (α-KG) analog and stabilizes HIF by inhibiting α-KG-dependent hydroxylases^43^. VH-298 blocks the interaction of VHL and HIF-α to stabilize HIF and upregulate HIF-target genes^44^. Both DMOG and VH-298 induced the HIF-1α under normoxic condition, with less induction magnitude as compared with hypoxic condition (Supplementary Fig. 10a). Under normoxic conditions, activation of HIF by treatment of DMOG and VH-298 can both induce dextran uptake and BSA digestion and the induced macropinocytosis can be abrogated by KO of EHD2 (Supplementary Fig. 10b, c). As HIF induces numerous target genes that are essential in different cellular processes, we over-expressed EHD2 in HIF-1α-KD cells (Supplementary Fig. 11a). OE of EHD2 restored macropinocytic capacity and BSA degradation in HIF-1α-KD cells under hypoxic conditions, indicating that EHD2 is the major HIF-1α target that promotes macropinocytosis in HCC cells (Supplementary Fig. 11b).

Since EHD2 directly interacts with actin filaments and is associated with endocytic activity, we postulated that EHD2 may promote actin-dependent membrane ruffle formation to initiate macropinocytosis. To analyze the membrane ruffle structures, we visualized polymerized actin (actin filaments) by immunofluorescence staining with phalloidin in EHD2-OE and EHD2-KO MHCC97L subclones. Membrane ruffles are protrusive structures located on the periphery or dorsal surface of cells characterized as upward-bending structures or backward-folding creases in the cell membrane^45,46^. For macropinocytosis, membrane ruffles are sheet-like extensions of the plasma membrane composed of an assembled network of actin filaments^17^. The membrane ruffles take the form of planar lamellipodia, circular cup-shaped membrane extensions (circular ruffles), or blebs^28^. Membrane ruffles occasionally curved into an open, crater-like cups of plasma membrane, named as “macropinocytic cup”^17^ (Supplementary Fig. 12a). OE of EHD2 promoted the formation of membrane ruffles (Fig. 4c), while KO of EHD2 reduced the membrane ruffles in MHCC97L cells (Supplementary Fig. 12b). EHD2 has been shown to interact with actin filament through binding of different partners as EHBP1 and NEK3^39,41^, we asked if the interactions of EHD2 and its binding partner are indispensable for initiation of macropinocytosis. We therefore knocked down EHBP1 or NEK3 in MHCC97L cells (Supplementary Fig. 13a, b). Interestingly, KD of EHBP1 but not NEK3 attenuated hypoxia-induced dextran uptake and BSA degradation in MHCC97L cells, indicating that the binding of EHD2 and EHBP1 may play a more important role in promoting actin rearrangement during membrane ruffling (Supplementary Fig. 13a, b). Next, we employed transmission electron microscopy (TEM) to visualize the structures of macropinosomes in EHD2-OE subclones. Strikingly, we could easily find macropinosomes with encapsulated particles in our EHD2-OE MHCC97L subclones (Fig. 4d). These structures matched the size of macropinosomes, which are 0.5–10 μm in diameter^28^.

Transferrin (Tf) is an iron-binding protein that enters the cells by binding with transferrin receptor (TfR) via clathrin-dependent endocytosis^47^. We found that in contrast to macropinocytosis, hypoxia reduced the transferrin uptake in HCC cells (Supplementary Fig. 13c). KO or OE of EHD2 has no effect on transferrin uptake (Supplementary Fig. 13c).

EHD2 has been reported to be important for fatty acid (FA) uptake^48^. To learn whether hypoxia-induced EHD2 is associated with FA uptake, we incubated MHCC97L-EHD2-KO/OE cells with green fluorescent-labeled fatty acid BODIPY™ FL C_12_. We found that hypoxia slightly induced FA uptake that could be abolished by KO of EHD2 (Supplementary Fig. 13d). Reversely, overexpression of EHD2 further promoted FA uptake under hypoxic condition (Supplementary Fig. 13d). Therefore, HIF-1/EHD2 axis might have multiple roles in metabolic adaptation.

**Fig. 5:**
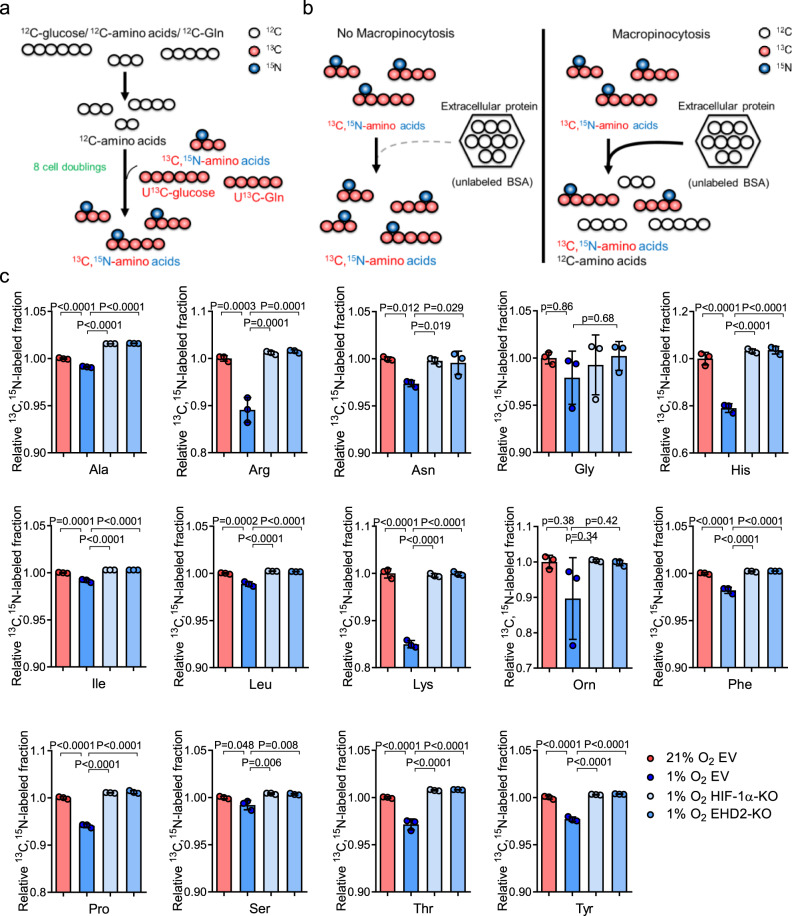
Integration of scavenged extracellular protein into the intracellular amino acid pool through hypoxia-induced macropinocytosis. **a**, **b** The workflow of stable isotope exchange and isotope-tracing experiment. **a** HCC cells were cultured in ^13^C,^15^N-DMEM containing U^13^C-glucose, U^13^C-glutamine (U^13^C-Gln), and ^13^C,^15^N-amino acids for 8 cell doublings. The unlabeled amino acids were replaced with isotope-labeled amino acids. **b** Isotope-labeled HCC cells were cultured in ^13^C,^15^N-DMEM with unlabeled BSA (^12^C-BSA). Without macropinocytosis, intracellular amino acids should remain to be isotope-labeled. With macropinocytosis, scavenged ^12^C-BSA becomes a source of amino acids and therefore decreases the ratio of isotope-labeled amino acids. **c** The ^13^C,^15^N-labeled fraction of amino acids in MHCC97L-EV, -HIF-1α-KO, and -EHD2-KO cells supplemented with ^12^C-BSA and exposed to 21 and 1% O_2_. Values were normalized to 21% O_2_ EV. Results were from 3 independent mass-spectrometry analysis. Error bars indicate mean ± SD. Two-way ANOVA with Bonferroni correction. Ala alanine, Arg arginine, Asn asparagine, Gly glycine, His histidine, Ile isoleukine, Leu leukine, Lys lysine, Orn ornithine, Phe phenylalanine, Pro proline, Ser serine, Thr threonine, Tyr tyrosine. Source data are provided as a Source Data file.

Thus far, we have provided evidence that the HIF-1/EHD2 pathway facilitates macropinocytosis and subsequent protein degradation in hypoxic HCC cells. We also showed that extracellular proteins rescue the growth of hypoxic HCC cells. A missing link is whether HIF-1/EHD2-mediated extracellular protein scavenging fuels hypoxic HCC cells metabolically to facilitate their growth in the context of O_2_ depletion. Metabolic incorporation of scavenged proteins was studied by prelabeling all amino acids in cells using stable isotope-containing medium, followed by treatment with unlabeled albumin (Fig. 5a). Since scavenged proteins would be the only source of unlabeled amino acids (^12^C-amino acids), macropinocytosis would decrease the ratio of isotope-labeled amino acids to total amino acids (Fig. 5b). MHCC97L-EV, MHCC97L-HIF-1α-KO, and MHCC97L-EHD2-KO cells were cultured in medium reconstituted with uniformly ^13^C-labeled glucose (U^13^C-glucose), ^13^C,^15^N-amino acids, and uniformly ^13^C-labeled glutamine (U^13^C-Gln) to replace intracellular amino acids with ^13^C,^15^N-amino acids (Fig. 5a and Supplementary Fig. 14). After 8 rounds of cell doubling, mass spectrometry confirmed that the efficiencies of isotope replacement for most amino acids reached over 90% (Supplementary Fig. 15). All the subclones were exposed to 21 and 1% O_2_ with isotope-labeled medium and unlabeled BSA (^12^C-BSA). Intriguingly, hypoxia markedly reduced the ratio of isotope-labeled amino acids to total amino acids (the ^13^C,^15^N-labeled fraction), which indicated metabolic incorporation of macropinocytosed proteins into the intracellular amino acid pool in the context of hypoxia (Fig. 5c). More interestingly, KO of HIF-1α or EHD2 restored the ^13^C,^15^N-labeled fraction, suggesting that the metabolic incorporation of scavenged proteins was dependent on the HIF-1/EHD2 pathway in HCC cells (Fig. 5c).

### EHD2 promotes HCC growth through induction of macropinocytosis in vivo

**Fig. 6:**
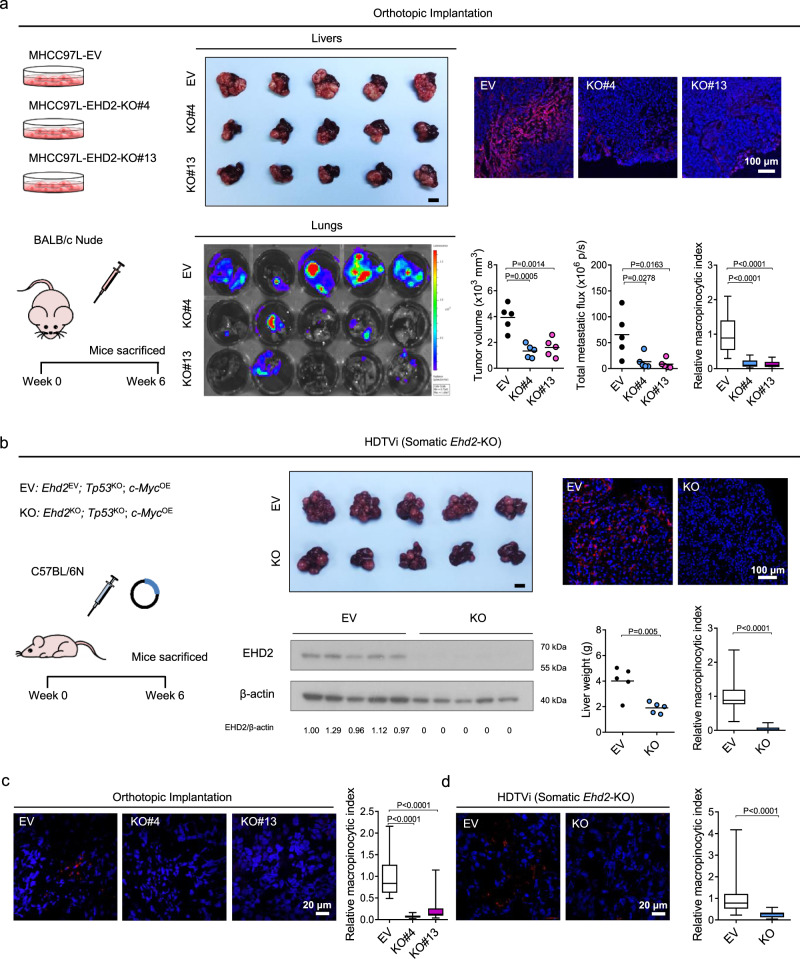
EHD2 promotes HCC growth and macropinocytosis in vivo. **a** Left: 6–8-week-old male nude mice were orthotopically implanted with luciferase-labeled MHCC97L-EV, -EHD2-KO#4, and -EHD2-KO#13 cells into the livers. HCCs were harvested 6 weeks after implantation. Middle: Livers (HCCs) harvested from the tumor-bearing mice (top) and bioluminescent imaging of lung metastases (bottom). Right: Confocal images demonstrate ex vivo dextran uptake/macropinosomes (red) and nuclei (DAPI, blue) in HCCs (top). Tumor volumes, bioluminescence of lung metastases, and macropinocytic indexes of HCC tissues were quantified (bottom). *n* = 5 biologically independent samples. **b** Left: HDTVi was performed in 8–10-week-old male wild-type C57BL/6 N mice to induce *Ehd2*^EV^; *Tp53*^KO^; *c-Myc*^OE^ (EV) and *Ehd2*^KO^; *Tp53*^KO^; *c-Myc*^OE^ (KO) HCCs. Middle: Image of livers (HCCs) was shown (top). Western blots demonstrate EHD2 protein expressions in EV and KO HCC tissues (bottom). Right: Confocal images show ex vivo dextran uptake/macropinosomes (red) and nuclei (DAPI, blue) in HCCs (top). Liver weights and macropinocytic indexes of HCCs (bottom). *n* = 5 biologically independent samples. **c** Confocal images show dextran uptake/macropinosomes (red) and nuclei (DAPI, blue) in orthotopic HCC tissues in nude mice. In vivo dextran labeling was performed by IV injection. *n* = 5 biologically independent samples. **d** Confocal images show dextran uptake/macropinosomes (red) and nuclei (DAPI, blue) in HCC tissues in HDTVi model by in vivo labeling of dextran through IV injection. Macropinocytic indexes were normalized to EV. *n* = 5 biologically independent samples. **a**, **b** Scale bars, 1 cm. The number of mice was represented by the number of dots. Scatter plot: center line, mean. Box-and-whisker: center line, median; box limits, 25th to 75th percentiles; whiskers, min to max. **a**, **c** One-way ANOVA with Bonferroni correction. **b**, **d** Two-tailed Student’s *t*-test. Source data are provided as a Source Data file.

**Fig. 7:**
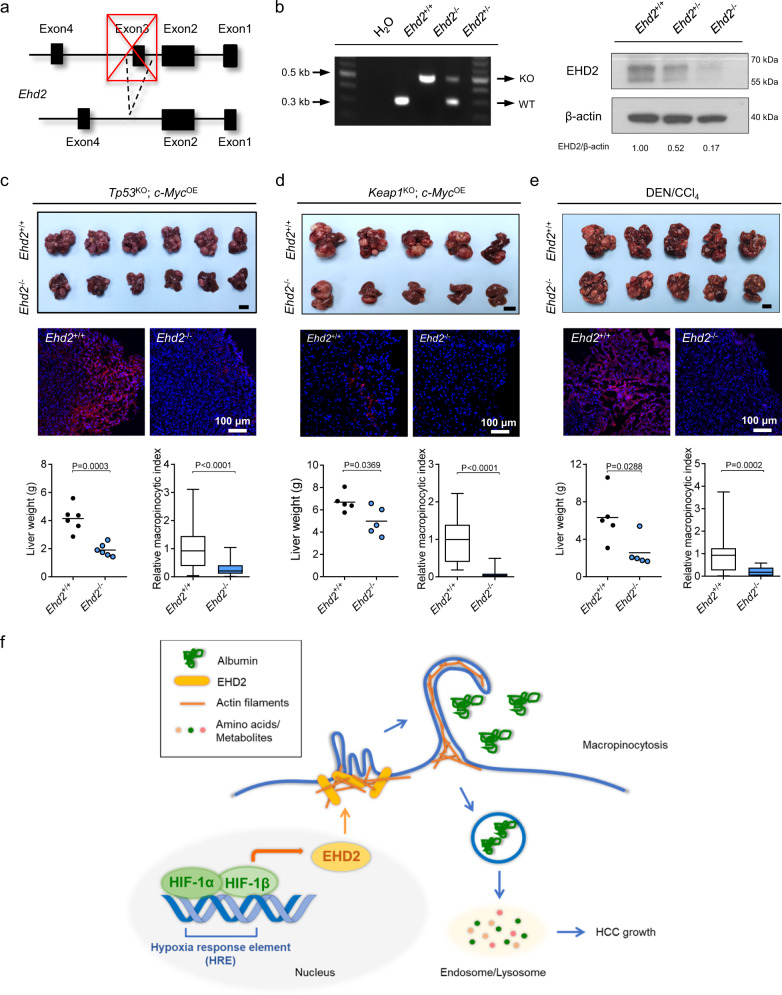
HIF-1/EHD2 induces macropinocytosis to support HCC growth in hypoxic conditions. **a** Transgenic strategy for generation of C57BL/6 N EHD2 germline KO (*Ehd2*^−/−^) mouse. Exon3 of mouse *Ehd2* was spliced in *Ehd2*^−/−^ mice. **b** Left: Gel picture shows PCR products from WT (*Ehd2*^+/+^) and KO (*Ehd2*^−/−^) alleles in *Ehd2*^+/+^, *Ehd2*^−/−^, and *Ehd2*^+/−^ mice. Right: Western blots demonstrate EHD2 expression in liver tissues from 8-week-old *Ehd2*^+/+^, *Ehd2*^+/−^, and *Ehd2*^−/−^ male mice. Results were representative for three independent experiments with similar results. **c** Top: Image of livers (*Tp53*^KO^; *c-Myc*^OE^ HCCs) from *Ehd2*^+/+^ and *Ehd2*^−/−^ mice. Middle: Confocal images show ex vivo dextran uptake/macropinosomes (red) and nuclei (DAPI, blue) in *Ehd2*^+/+^ and *Ehd2*^−/−^ HCC tissues. Bottom: Liver weights and macropinocytic indexes from *Ehd2*^+/+^ and *Ehd2*^−/−^ mice. *n* = 6 biologically independent samples. **d** Top: Image of livers (*Keap1*^KO^; *c-Myc*^OE^ HCCs) from *Ehd2*^+/+^ and *Ehd2*^−/−^ mice. Middle: Confocal images show ex vivo dextran uptake/macropinosomes (red) and nuclei (DAPI, blue). Bottom: Liver weights and macropinocytic indexes from *Ehd2*^+/+^ and *Ehd2*^−/−^ mice. *n* = 5 biologically independent samples. **e** Top: Image of livers (DEN/CCl_4_ HCCs) was shown. Middle: Confocal images demonstrate ex vivo dextran uptake/macropinosomes (red) and nuclei (DAPI, blue) in HCC tissues from *Ehd2*^+/+^ and *Ehd2*^−/−^ mice. Bottom: Liver weights and macropinocytic indexes from *Ehd2*^+/+^ and *Ehd2*^−/−^ mice. *n* = 5 biologically independent samples. **f** Research summary. Hypoxia stabilizes HIF-1 that transcriptionally activates EHD2. EHD2 interacts with actin filaments to promote membrane ruffling that initiates macropinocytosis in hypoxic HCC cells. Hypoxia-induced macropinocytosis promotes the engulfment of extracellular proteins (e.g., albumin). Degraded extracellular proteins are integrated into the intracellular amino acid pool to support HCC cell growth under hypoxic conditions. **c**–**e** Macropinocytic indexes were normalized to *Ehd2*^+/+^. Scale bars, 1 cm. The number of mice was represented by the number of dots. Scatter plot: center line, mean. Box-and-whisker: center line, median; box limits, 25th to 75th percentiles; whiskers, min to max. Two-tailed Student’s *t*-test. Source data are provided as a Source Data file.

To elucidate the roles of EHD2 in macropinocytosis and HCC in vivo, we employed a great variety of mouse HCC models. First, we orthotopically implanted luciferase-labeled MHCC97L-EV or MHCC97L-EHD2-KO cells into the liver of nude mice. KO of EHD2 in MHCC97L cells dramatically suppressed HCC tumor growth, lung metastasis, and macropinocytosis (Fig. 6a). To investigate the correlation of hypoxia/HIF and dextran uptake in vivo, we performed in vivo dextran labeling together with a widely employed hypoxic marker pimonidazole and costained the tumors with GLUT1. Pimonidazole is a 2-nitroimidazole compound that can covalently bind to the thiol-containing proteins in hypoxic cells^49^. We generally observed a positive correlation of higher TMR-dextran uptake with higher pimonidazole staining and GLUT1 staining in the tumors (Supplementary Fig. 16a). However, the patterns for various hypoxic markers and TMR-dextran labeling do not completely overlap. In EHD2-KO tumors, the level of dextran labeling was significantly decreased, while the pimonidazole and GLUT1 signals reduced slightly (Fig. 6a and Supplementary Fig. 16a). Second, by HDTVi, we delivered a CRISPR–Cas9 system with two linked sgRNAs to knock out *Tp53* and *Ehd2* and a Sleeping Beauty transposon system to overexpress *c-Myc* somatically. Somatic KO of *Ehd2* drastically repressed the growth and macropinocytic capability of *Tp53*^KO^; *c-Myc*^OE^ HCC (Fig. 6b). To obtain direct evidence that the macropinocytosis is induced in HCC tissue, we injected dextran through tail vein and collected tumor and nontumorous tissues to evaluate the dextran uptake capacity as a direct indicator of macropinocytosis level in vivo. In both orthotopic and HDTVi models, tumor tissue demonstrated a higher level of dextran labeling, while nontumorous tissue was barely labeled with dextran (Fig. 6c, d and Supplementary Fig. 16b, c). The macropinocytic capacity was suppressed in EHD2-KO clones in the orthotopic implantation model and somatic *Ehd2* KO tumors in HDTVi model in vivo (Fig. 6c, d). Third, we established a transgenic *Ehd2*-KO (*Ehd2*^−/−^) mouse line (Fig. 7a, b). *Ehd2*-KO mice (*Ehd2*^−/−^) exhibited no obvious defects during their normal life span. Similar to somatic KO of *Ehd2*, germline KO of *Ehd2* repressed the growth and macropinocytosis of *Tp53*^KO^; *c-Myc*^OE^ HCC and *Keap1*^KO^; *c-Myc*^OE^ HCC, which were induced by HDTVi (Fig. 7c, d and Supplementary Fig. 17a, c). Since human HCC is often preceded by fibrosis, we induced HCC in *Ehd2*-KO mice with the classic method involving hepatocarcinogen diethylnitrosamine (DEN) and fibrotic agent carbon tetrachloride (CCl_4_) exposure. Consistent with all other mouse HCC models described above, *Ehd2*-KO (*Ehd2*^−/−^) mice had smaller tumors with a weaker macropinocytic capability with the fibrotic background than *Ehd2*-wild-type (WT) mice (*Ehd2*^+/+^) (Fig. 7e and Supplementary Fig. 17e). These findings strongly indicate that EHD2-mediated macropinocytosis is not restricted to any specific oncogenic mutations but rather is a general phenomenon in HCC. Notably, the general immune landscape, as reflected by the number of tumor-infiltrated immune cells, including CD4^+^ T cells, CD8^+^ T cells, macrophages, myeloid-derived suppressor cells (MDSCs), and dendritic cells (DCs), was mostly unaffected in *Ehd2*^−/−^ mice (Supplementary Fig. 17b, d, f), suggesting that germline deletion of *Ehd2* mainly affects cancer cells but does not have an apparent impact on the immune microenvironment of HCC. Multiple models demonstrated the significant role of EHD2 in HCC growth in vivo. Interestingly, we found that KO of EHD2 did not affect proliferation of hypoxic HCC cells in vitro under nutrient-replete conditions. However, KO of EHD2 significantly reduced proliferation of hypoxic HCC cells under nutrient-insufficient condition (10% amino acid comparing with normal culture media, 10% AA) in the presence of BSA (Supplementary Fig. 18a). Consistently, OE of EHD2 did not affect proliferation of hypoxic HCC cells but significantly increased proliferation of hypoxic HCC cells under nutrient-insufficient condition in the presence of BSA (Supplementary Fig. 18b). Effects were less prominent in the absence of BSA (Supplementary Fig. 18c). The tumor vasculature is mostly abnormal and dysfunctional, which limits the delivery of nutrients. As macropinocytosis provides an alternative fuel to cancer cells during nutrient limitation, it is not surprising that a relatively prominent effect of EHD2 could be observed in vivo.

## Discussion

Macropinocytosis is an important cellular process that allows cancer cells to scavenge extracellular proteins or necrotic cell debris to use as fuels in nutrient-limiting conditions. Tumors outstrip the vasculature in growth speed, leading to deprivation of nutrients such as glucose, AAs, and O_2_. Our study reveals a regulatory mechanism by which hypoxic cancer cells induce protein scavenging through macropinocytosis. Using HCC models, we show that hypoxic cancer cells (1% O_2_) have a higher macropinocytic capability than normoxic cancer cells (21% O_2_). Furthermore, we show that HIF-1 induces the transcription of EHD2, driving actin-dependent membrane-ruffle formation and macropinocytosis in hypoxic cancer cells. Germline or somatic KO of *Ehd2* in mice significantly represses macropinocytosis and growth of HCC. Inhibitors blocking HIF or membrane ruffle formation significantly represses macropinocytosis and the development of HCC. Moreover, we demonstrate that treatment of PHD and VHL inhibitor can both induce dextran uptake and BSA digestion under normoxic conditions. Using pimonidazole and GLUT1 as hypoxic markers in HCC tissues, we generally observed an overlapping pattern of pimonidazole staining, GLUT1 staining, and dextran uptake in the tumors. In EHD2-KO tumors, the level of dextran labeling is significantly decreased, while the pimonidazole and GLUT1 signals only reduced slightly. We also found that the dextran uptake can also be observed in some regions with less intensity of hypoxic markers in the in vivo model, indicating that there might be HIF-independent mechanism in macropinocytosis. Compared with the in vitro experiment in which we directly cultured the HCC cells in hypoxia, intratumoral hypoxia cannot be as precisely defined in vivo but generally indicated by various hypoxic markers. Therefore, more convincing evidence to directly confirm the induction of macropinocytic index in hypoxic tumors could be achieved when coupled with analysis from advanced technologies such as spatial transcriptomics to better define hypoxic regions.

Most studies on macropinocytosis in cancer focus on tumors with oncogenic *KRAS* mutations. *KRAS* is frequently mutated and activated in cancers such as pancreatic and lung cancers. However, *KRAS* mutations are not frequent in liver cancer. On the other hand, the oncogene *C-MYC* is overexpressed in up to 40–60% of early HCC cases, as indicated by genomic amplification of the 8q-22–24 region^50^. In addition, frequent mutations are found in several pathways in HCC, including the telomere maintenance (TERT), cell cycle (TP53), self-renewal/stemness (APC/AXIN/β-catenin), oxidative stress (KEAP1/NRF2), epigenetic modification (ARID1A/ARID1B/ARID2), and proliferation (TSC/PTEN) pathways^51^. To simulate the genetic makeup of human HCC, we established *Tp53*^KO^; *c-Myc*^OE^ and *Keap1*^KO^; *c-Myc*^OE^ mouse HCC models by HDTVi as a proof-of-concept. We found that germline or somatic KO of *Ehd2* significantly impeded the progression of both *Tp53*^KO^; *c-Myc*^OE^ and *Keap1*^KO^; *c-Myc*^OE^ mouse HCCs. In addition, germline or somatic KO of *Ehd2* impeded the growth of hepatocarcinogen-induced HCC. Interestingly, although HCC cell lines such as MHCC97L, PLC/PRF/5, and Hep3B have mutations in different pathways^52,53^, macropinocytic activity was consistently induced by hypoxia. Our data consistently supported the role of HIF-1/EHD2-induced micropinocytosis, regardless of the genetic background of the model. Therefore, our data suggest that apart from the oncogenic RAS pathway, the HIF-1/EHD2 pathway also plays a crucial role in promoting macropinocytosis in tumors.

Our current study demonstrates that EHD2 is the transcriptional target of HIF-1 that mediates membrane ruffle formation and subsequent macropinocytosis. In fact, EHD2 has been shown to participate in other endocytic processes, such as caveolae-mediated and clathrin-mediated endocytosis, by stabilizing caveolin and clathrin at the membrane to ensure the successful formation of membrane vesicles^40,41^. Hypoxia and HIFs induce caveolae formation in HCC^54^. However, dextran uptake was not affected when we knocked down caveolin1 or clathrin components, while transferrin uptake was not affected by KO or OE of EHD2, suggesting that our observation on hypoxia-induced macropinocytosis is independent of caveolae- and clathrin-mediated endocytosis. Our study also implies that HIFs/EHD2 may be a central regulatory pathway that controls endocytosis in hypoxic cells. To date, the roles of EHD2 have been mostly demonstrated in noncancerous cells, such as fibroblasts. High EHD2 mRNA expression correlates with aggressive features in papillary thyroid carcinoma, such as extrathyroidal extension, advanced tumor stages, lymph-node metastasis, and a relatively high recurrence rate^55^. Our in-house cohort data together with TCGA database demonstrated overexpression of EHD2 in HCC. Although multiple stromal cells exist in the HCC tumor microenvironment, the majority cells within HCC represent liver cancer cells^56^. Besides, we have observed that EHD2 expression was significantly induced by hypoxia in HCC cells. Our in vitro and in vivo dextran labeling both show that hypoxia induced macropinocytosis in HCC cells or tissues but not in normal liver, highlighting the role of EHD2 in macropinocytosis in hypoxic HCC. Our study draws the first link regarding the role of EHD2 in macropinocytosis-mediated metabolic adaptation. More translational studies should focus on the identification of specific EHD2 inhibitors in the future.

Although macropinocytosis was reported as a general phenomenon in cancer in 1937^57^, the underlying regulatory mechanisms remain largely elusive. In the past few years, studies have revealed that macropinocytosis in cancer could be induced by RAS activation^23,26^, mTORC inhibition^30,58^, PI3K activation^59^, AMPK activation^21^, PTEN deletion^21^, EGFR–Pak activation^60^, and NRF2 activation^61^. Activated KRAS was demonstrated to be a crucial regulator of macropinocytosis in a mouse pancreatic cancer model^26^. This study showed that albumin in the circulation is internalized, catabolized, and utilized by RAS-transformed pancreatic cancer cells^26^. Macropinocytosis, as opposed to protein anabolism, is counteracted by mTORC1^30,58^. mTORC1 is important for translation and *de novo* protein synthesis when amino acids are sufficient. In an AA-depleted environment, mTORC1 activity is initially reduced. However, when cells start to catabolize engulfed albumin in lysosomes, mTORC1 is recruited to the lysosomes and becomes activated^30^. Partial mTORC1 inhibition reserves the limited AAs derived from macropinocytosis, favors the growth of macropinocytosis-dependent cancer cells, and enhances the growth of KRAS-induced pancreatic cancer^58^. PI3K converts phosphatidylinositol (4,5)-bisphosphate (PIP2) into phosphatidylinositol (3,4,5)-trisphosphate (PIP3), an important lipid at the cell membrane, to close the macropinosome^59^. As shown in macrophages, PI3K activation facilitates actin-dependent membrane-ruffle formation and macropinocytosis^59^. PI3K is counteracted by PTEN, which is frequently deleted in many cancer types. Prostate cancer cells with PTEN loss rely on AMPK activation-mediated macropinocytosis under nutrient-insufficient conditions^21^. A recent finding demonstrated that macropinocytosis could be specifically induced in a subset of pancreatic cancer cell lines with low intrinsic macropinocytic activity by glutamine depletion through the activation of EGFR–PAK signaling^60^. Resonating this study, our finding also suggests that macropinocytosis could be specifically induced by hypoxia. Coincidentally, glutamine and O_2_ are both depleted in the tumor cores due to insufficient blood supply. Abnormal tumor vascularization is likely associated with limited nutrient supply that drives macropinocytosis. Since our experiments are mostly performed in the presence of essential amino acids and nonessential amino acids (including glutamine) as well as glucose, our findings provided evidence that the HIF-1/EHD2-mediated macropinocytosis is independent of the amino acid availability. Interestingly, Lee S et al. and our current study corroborated to show that deprivation of specific nutrients such as glutamine and O_2_ respectively could independently turn on macropinocytosis^60^.

Macropinocytosis highly resembles autophagy, which allows cells to degrade intracellular organelles to generate proteins that sustain growth under nutrient-depleted conditions. An elegant study showed that hypoxia/HIF-1 induces autophagy by transcriptionally activating BNIP3^62^. Interestingly, a recent finding suggested that NRF2 mediates macropinocytosis in cells that fail to undergo autophagy through activating macropinocytosis-related genes such as SDC1, NHE1, CDC42, and PI3KCG^61^. Co-blockade of autophagy and macropinocytosis could synergistically suppress the growth of pancreatic cancer in xenograft model, suggesting a complementary role of autophagy and macropinocytosis in supporting cancer growth^61^. Meanwhile, our current study demonstrates that hypoxia induces macropinocytosis through HIF-1, which transcriptionally activates EHD2 to mediate membrane ruffle formation to initiate macropinocytosis. O_2_ availability in all HCC tumors is typically inadequate due to poor blood perfusion. HIFs are evolutionarily conserved transcription factors that regulate the transcriptional response in low O_2_ conditions. Our study showed that HIFs are central regulators that help hypoxic cells adapt to nutrient limitations. By unprecedentedly illustrating the role of HIFs in macropinocytosis, our study has once again demonstrated the ever-expanding roles of HIFs in tumor biology.

Apart from macropinocytosis, cell-surface receptors such as cubilin and megalin can be used to internalize albumin^63^. After being internalized together with its receptor, albumin is either sent for lysosomal degradation or conjugated with neonatal Fc receptor (FcRn) and exocytosed^63^. Human Protein Atlas confirmed that liver and liver cancer tissues do not express the albumin receptors (Supplementary Fig. 19). Furthermore, we used 70 kDa dextran as an indicator to specifically evaluate the macropinocytic capacity of cells but not albumin receptor-induced internalization. Consistent blockade of dextran uptake was observed when HCC cells were treated with HIF inhibitor digoxin as well as macropinocytosis inhibitors EIPA and IPA-3. Because these inhibitors have a broad spectrum of targets, we also validated our results in our HIF-1 or EHD2 KD/KO HCC cells to demonstrate that hypoxia-induced dextran uptake was mediated through HIF-1/EHD2 pathway specifically. Furthermore, overexpression of EHD2 could restore macropinocytosis in HIF-1 KD HCC cells.

All these pieces of evidence lend credence to our observation that hypoxia-induced albumin uptake in our experimental models occurs through macropinocytosis. Albumin is known to be a lipid carrier in the circulation^64^. Notably, reports have shown that hypoxic cancer cells take up and metabolize extracellular lipoproteins as a lipid source^65^. HCC cells also take up circulating free fatty acids through the fatty acid translocase CD36^66^. Cancer cells with mutated RAS resemble hypoxic cells that scavenge serum fatty acids^65^. Our study mainly focuses on the use of albumin as a source of amino acids. Whether lipids scavenged through the albumin carrier can be used as a source of lipids needs to be explored in the future.

Recent research has established a link between macropinocytosis and cancer therapeutic resistance. Macropinocytosis enables pancreatic cancer cells with activated KRAS and deficient PTEN to overcome mTOR inhibitor resistance^67^. The lysosomal inhibitor CQ sensitizes pancreatic cancer cells to an mTOR inhibitor by suppressing the degradation of macropinocytosed proteins^67^. Apart from albumin and other extracellular proteins, necrotic cancer cell debris can also be taken up and utilized as a nutrient source by cancer cells through macropinocytosis^20,21^. Chemotherapies and radiotherapies induce necrosis in cancer cells, providing a rich nutrient source for macropinocytic cancer cells. Macropinocytosis, therefore, provides a general mechanism of resistance to any necrosis-inducing standard systemic cancer therapy^20^. Depletion of nutrients, including O_2_, is a major cause of tissue necrosis. A large body of literature has demonstrated the obligatory relationship between tissue ischemia and necrosis. Histological analysis of breast cancer tissue specimens showed that 90% of necrotic-cancer regions express at least one standard indicator of hypoxia, such as HIF-1, CA9, or GLUT1^68^. It is plausible that cell debris generated via hypoxia-induced necrosis provides an additional extracellular nutrient source for HCC cells to scavenge through macropinocytosis. As a proof-of-concept, our study mainly used albumin as the scavenging nutrient to evaluate the macropinocytic capabilities of different HCC subclones. However, our findings on the roles of HIF-1/EHD2 in membrane ruffling and macropinocytosis are not limited to albumin and could be extended to other cellular proteins and necrotic cell debris.

Our study has provided important translational insights. First, macropinocytosis has never been demonstrated previously in a liver cancer model, and our study highlighted the metabolic significance of macropinocytosis in the survival of HCC cells. We showed that the HIF inhibitor digoxin significantly repressed macropinocytosis and the growth of HCC. Notably, like many other macropinocytosis inhibitors, HIF inhibitors have pleiotropic effects on HCC growth, given that HIF has diverse protumorigenic functions. We also showed that IPA-3, an inhibitor that targets PAK1, which is a downstream target of RAC1 responsible for actin-mediated membrane ruffling^28^, also effectively perturbed the growth of HCC by blocking macropinosome formation. In addition to HIF and PAK1 inhibitors, lysosomal inhibitors also suppressed hypoxia-induced macropinocytosis in HCC cells through blockade of protein digestion. While more specific macropinocytosis inhibitors have yet to be developed, our study suggests that inhibitors targeting various steps in macropinocytosis could be exploited as new therapies against HCC, which to date, has no effective molecular targets. Second, we showed that hypoxic cancer cells have a propensity to undergo macropinocytosis. Albumin-conjugated paclitaxel (Abraxane^®^), which was approved by the FDA for treating metastatic breast, non-small-cell lung, and pancreatic cancers, is expected to be effectively taken up by hypoxic HCC cells. In other words, albumin-conjugated chemotherapeutic drugs or targeted therapies might be used to specifically target hypoxic cancers.

In conclusion, our study has provided mechanistic and translational insights into HCC that could be applied to other solid cancers with hypoxia. We identified the precise mechanism by which the HIF/EHD2 pathway supports macropinocytosis-mediated metabolic reprogramming in cancer, providing an important molecular basis for the design of innovative cancer-therapeutic strategies against macropinocytosis.

## Methods

### Ethical regulations

This study complies with all relevant ethical regulations. All animal experiments and study protocols were approved by the Committee on the Use of Live Animals in Teaching and Research (CULATR) of the University of Hong Kong according to the Animals (Control of Experiments) Ordinance of Hong Kong. Prior approval for the use of clinical tissue samples was acquired from the Institutional Review Board of The University of Hong Kong and the Hospital Authority of Hong Kong. The patients signed consent forms to acknowledge the use of their resected tissues for research purposes.

### Cell culture

Human HCC cell line MHCC97L was a gift from Dr. Z.Y. Yang of Fudan University (Shanghai, China). Human HCC cell lines PLC/PRF/5, Hep3B, and immortalized liver cell lines MIHA and THLE3 were purchased from American Type Culture Collection (ATCC). All cells were cultured in Dulbecco’s Modified Eagle Medium–high-glucose (DMEM–HG) media (12800017, Gibco) supplemented with 1 mM sodium pyruvate, 1% penicillin–streptomycin (Pen–Strep) (15140122, Gibco), and 10% fetal bovine serum (FBS) (10270106, Gibco). Cells were maintained in a 37 °C incubator under 5% CO_2_. All cell lines were authenticated with the AuthentiFiler^TM^ PCR Amplification Kit (4322288, Applied Biosystems^TM^). Mycoplasma screening is performed every 1–2 months for all cell lines used and our cells are mycoplasma-free.

### Establishment of stable HCC cell lines

MHCC97L-HIF-1α-KO#12, MHCC97L-HIF-1α-KO#32, MHCC97L-shHIF-1α, and MHCC97L-shHIF-2α cells were generated as described^69^. Stable EHD2-KO/HIF-1α-KO cells were generated using the CRISPR–Cas9 system. Single-guide RNAs (sgRNAs) targeting the human *EHD2/HIF-1A* gene were inserted into the lentiGuide-Puro plasmid. Viral particles were generated by pPACKH1 HIV Lentivector Packaging Kit (LV500A-1, SBI) using the lentiviral system and infected into Cas9-expressing MHCC97L cells. Stable KO clones and empty-vector (EV)-expressing cells were selected using puromycin (P7255, Sigma). Stable EHD2-OE cells were generated using the CRISPR/dCAS9 Synergistic Activation Mediator-activating system as described^70^. Viral particles containing sgRNAs targeting *EHD2* promoter were generated and infected into dCas9-Vp64 and MS2-p65-HSF1-expressing MHCC97L or Hep3B cells. Stable OE clones and EV-expressing cells were selected using zeocin (R25001, Invitrogen).

For knockdown (KD) clones, short-hairpin RNAs (shRNAs) targeting human *EHD2/CAV1/CLTC/EHBP1/NEK3* genes were expressed in pLKO.1-puro vectors. Viral particles were generated using the lentiviral system and infected into parental PLC/PRF/5 or MHCC97L cells. Stable KD and non-target-control (NTC) cells were selected using puromycin.

For HIF-1α-KD-EHD2-OE clones, MHCC97L cells were first infected with viral particles carrying empty vector (EV) or shRNA targeting human *HIF-1A* gene. Stable HIF-1α-KD clone was selected using puromycin. After validation of successful KD of HIF-1α, EHD2-OE stable subclone was established using the CRISPR/dCAS9 Synergistic Activation Mediator-activating system in MHCC97L-HIF-1α-KD cells as described^70^.

All sgRNA and shRNA target sequences are provided in Supplementary Table 1.

### Reagents and drugs

Glutamine-depleted (-Gln), essential amino acid-depleted (-EAA), nonessential amino acid-depleted (-NEAA), low-glutamine (0.2 mM glutamine, 0.2 Q), and 10% amino acid (10% AA) DMEM media were prepared from the base DMEM medium (D9800-26, US Biologics). For 10% AA-DMEM, 10% of amino acid concentrations of standard DMEM formula were added. For other nutrient-deficient media, essential amino acid mixture (M5550, Sigma-Aldrich), nonessential amino acid mixture (M7145, Sigma-Aldrich), sodium pyruvate, and Pen–Strep were added at a concentration equivalent to the formula of standard DMEM. Bovine serum albumin (BSA) (A4918, Sigma-Aldrich) was reconstituted in PBS. EIPA (A3085, Sigma-Aldrich), BafA1 (19–148, Sigma-Aldrich), CQ (C6628, Sigma-Aldrich), IPA-3 (I2285, Sigma-Aldrich), and VH-298 (SML1896, Sigma-Aldrich) were dissolved in dimethyl sulfoxide (DMSO). DMOG (D5193, Sigma-Aldrich) was reconstituted in ultrapure water (10977015, Gibco). Pimonidazole (HP2-100 Kit, Hypoxyprobe, Inc.) was reconstituted in saline. 80 mg/kg pimonidazole was injected intraperitoneally 1.5 h before tumor harvest. For in vivo treatment, IPA-3 was dissolved in 10% DMSO in β-captisol. Mice were treated with 2 mg/kg IPA-3 biweekly i.p. or 1.2 mg/kg/day i.p. digoxin (Aspen Pharmacare Australia) daily or vehicle controls through intraperitoneal injection.

### In vitro macropinocytic assay

Normal liver and HCC cells were seeded on coverslips in DMEM with 10% FBS. Cells were pre-exposed to 21 and 1% O_2_ for 36 h and serum-starved for 12 h. Dig, EIPA, IPA-3, BafA1, CQ, DMOG, and VH-298 were pretreated at the time of serum removal. For TMR-dextran labeling, cells were incubated with 1 mg/mL 70 kDa tetramethylrhodamine–dextran (D1818, Invitrogen) in serum-free medium and exposed to 21 and 1% O_2_ for 6 min. For dextran and dye-quenched/constitutively fluorescent BSA dual labeling, cells were incubated with 0.2 mg/mL TMR dextran and 0.08 mg/mL dye-quenched FITC-conjugated BSA (D12050, DQ^TM^ Green BSA, DQ-FITC-BSA, Invitrogen) or constitutively fluorescent BSA (A23015, FITC-BSA, Alexa Fluor™ 488 conjugate, Invitrogen) in serum-free DMEM for 1.5 h at 21 and 1% O_2_. Labeling was stopped by ice-chilling and washing with PBS. Cells were fixed with 4% formalin and mounted with fluoroshield mounting medium with DAPI (Abcam). Imaging was performed using LSM 780/800/900/980 confocal microscopes (Carl Zeiss). Data were analyzed by ZEN 3.3 and ImageJ (64-bit Java 1.8.0_172) software. Cell boundaries were defined by the bright-field imaging captured through TPMT detector overlaid with fluorescent channels. At least 200 cells were captured randomly for analysis. The analysis for macropinocytic index and DQ-FITC-BSA/FITC-BSA particle area was not blinded.

Macropinocytic index = total TMR-dextran fluorescent particle area/total cell area × 100

DQ-FITC-BSA/FITC-BSA particle area = total DQ-FITC-BSA/FITC-BSA fluorescent particle area/total cell area × 100

### Ex vivo macropinocytic assay

Freshly harvested tumor tissues were diced into 2 mm^3^ and washed with PBS. Tumor tissues were incubated with 1 mg/mL TMR dextran diluted in serum-free medium at 37 °C for 30 min. Tissues were washed with PBS, fixed in 4% formalin, and snap-frozen in liquid nitrogen. Frozen sections were mounted with fluoroshield mounting medium with DAPI (ab104139, Abcam). Confocal images were captured by LSM 780/800 confocal microscopes (Carl Zeiss). Data were analyzed by ZEN 3.3 and ImageJ (64-bit Java 1.8.0_172) software. For each sample, 4–5 random areas were captured for analysis.

Ex vivo macropinocytic index = total TMR-dextran fluorescent particle area/total tissue area × 100

### In vivo macropinocytic assay

In the orthotopic implantation and HDTVi model, mice were IV injected with 1 mg of TMR-dextran dissolved in saline 1 h before tissue harvest. Tumor and nontumorous tissues were collected and snap-frozen in liquid nitrogen. Frozen sections were mounted with fluoroshield mounting medium with DAPI. Five tumors from each experimental group were analyzed. For each sample, 5 random areas were captured by LSM 900/980 confocal microscopes (Carl Zeiss). Data were analyzed by ZEN 3.3 and ImageJ (64-bit Java 1.8.0_172) software. Total tissue area was determined by bright-field imaging captured through TPMT detector coupled with fluorescent channel.

In vivo macropinocytic index = total TMR-dextran fluorescent particle area/total tissue area × 100

### RNA extraction, reverse transcription, and real-time quantitative PCR

Total RNA was extracted by TRIzol^®^ reagent (15596018, Life Technologies) according to the manufacturer’s protocol. Reverse transcription was carried out using MultiScribe^TM^ Reverse Transcriptase (4311235, Invitrogen) and cDNA was used for real-time quantitative PCR analysis (RT-qPCR). Taqman^®^ Gene Expression Assay (4304437, Applied Biosystems^TM^) and SYBR^TM^ Green qPCR Master Mix (4367659, Applied Biosystems^TM^) were used to evaluate the mRNA expressions of *EHD1/EHD2/EHD3/EHD4/CAV1/CLTC/HIF-1A/EHBP1/NEK3* and housekeeping gene 18S in human specimens (Taqman^®^) and cell lines (SYBR^TM^ Green). RT-qPCR was carried out by StepOnePlus™ Fast Real-Time PCR System (Applied Biosystems^TM^). Primer sequences are provided in Supplementary Table 2.

### Protein extraction and western blotting

Total human HCC cell lysate was extracted using RIPA lysis buffer with cOmplete^TM^ protease inhibitor (4693132001, Roche Diagnostics) and PhosSTOP phosphatase inhibitor (4906845001, Roche Diagnostics). Mouse tumor tissues were snap-frozen and homogenized in RIPA lysis buffer. HIF-1α antibody (#3716, Cell Signaling Technology), HIF-2α antibody (ab199, Abcam), EHD2 antibodies (PA549403 [Thermo], 154784 [Abcam]), and β-actin antibody (A5316, Sigma-Aldrich) were used for immunoblotting. Band intensities for HIF-1α or EHD2 expression were analyzed using ImageJ and were normalized to band intensities of β-actin.

### Cell proliferation assay

For cell-counting assay, 3 × 10^4^ cells were seeded on 12-well plates in triplicates. Cells were exposed to 21 and 1% O_2_ after cell seeding. Cells were counted at different time points (24, 48, 72, and 96 h) using TC20^TM^ Automated Cell Counter (Bio-Rad). For XTT and BrdU assays, 1 × 10^3^ cells were seeded on a 96-well culture plate in triplicates. Cell proliferations were measured 5 days after being cultured in 21 and 1% O_2_ conditions. For BrdU assay, cells were prelabeled with BrdU labeling solution (Cell Proliferation ELISA, BrdU, colorimetric) (11647229001, Roche) 24 h before measurement. Cells were fixed by FixDenat and incubated in Anti-BrdU-POD working solution. After incubation, the antibody conjugate was removed completely by tapping and cells were washed with PBS. Cells were incubated with substrate solution and the absorbance was measured by multifunctional plate reader (TECAN SPARK) at 370 nm (reference wavelength 492 nm). For XTT assay, proliferating cells were labeled with cell proliferation kit II (3-bis-(2-methoxy-4-nitro-5-sulfophenyl)-2H-tetrazolium-5-carboxanilide (XTT)) (11465015001, Roche) according to the manufacturer’s protocol.

### Cell death assay

HCC cells were exposed to 21 and 1% O_2_ for 5 days. Cells were collected by trypsinization and incubated with 30 nM SYTOX™ Green Dead Cell Stain (S34860, Invitrogen) for 40 min in the dark. Dead cell populations were analyzed by flow cytometry by BD LSRFortessa^TM^ flow cytometer (BD Biosciences) and the percentage of dead cell (SYTOX Green+, SG+) was calculated using FlowJo v10.7 software (FlowJo, LLC).

### The Cancer Genome Atlas

EHD2 mRNA-expression values in human HCC, ESCA, KIRC, ICC, and their corresponding NT tissues were retrieved from the transcriptome sequencing data available at the Cancer Genome Atlas (TCGA) (cBioportal for Cancer Genomics: http://www.cbioportal.org/).

### The Human Protein Atlas

Megalin (LRP2) and cubilin (CUBN) expressions in human tissues and cancers were retrieved from immunohistochemistry data available from the Tissue Atlas and the Pathology Atlas, respectively, at The Human Protein Atlas (LRP2: https://www.proteinatlas.org/ENSG00000081479-LRP2; CUBN: https://www.proteinatlas.org/ENSG00000107611-CUBN).

### Clinical tissue samples

Human HCC tissues and corresponding NT tissues were harvested by surgical resections from HCC patients admitted to QMH-HKU. The tissues were snap-frozen in liquid nitrogen and stored at −80 °C. Prior approval for the use of clinical tissue samples was acquired from the Institutional Review Board of The University of Hong Kong and the Hospital Authority of Hong Kong. The patients signed consent forms to acknowledge the use of their resected tissues for research purposes.

### Clinicopathological correlation

Correlation studies on the expression level of EHD2 with different clinicopathological parameters of the HCC patients such as tumor size, pTMN stage, cellular differentiation by Edmondson grading, direct tumor invasion, tumor microsatellite formation, venous invasion, and tumor encapsulation were performed using SPSS 20.0 software.

### Chromatin immunoprecipitation

HCC cells exposed to 21 and 1% O_2_ were fixed with 4% formaldehyde and sonicated. Sheared DNA fragments were immunoprecipitated with HIF-1α (ab1, Abcam), HIF-1β (ab2, Abcam), and IgG control (sc-2027, sc-2762, Santa Cruz) using protein A/salmon-sperm DNA agarose beads (16–157, Merck Millipore). The DNA–protein–antibody complex was washed sequentially with different gradients of salt buffers and eluted in 1% SDS/0.1 M NaHCO_3_. ChIP DNA was analyzed by RT-qPCR with primers targeting HRE identified in the *EHD2* promotor. Primer sequences are provided in Supplementary Table 2.

### Phalloidin staining

HCC cells were seeded onto glass coverslips and exposed to 21 and 1% O_2_ for 48 h. Cells were washed with PBS and fixed with 4% formaldehyde at room temperature for 30 min. Cells were incubated with 0.05 μg/mL phalloidin–tetramethylrhodamine–B isothiocyanate (P1951, Phalloidin-TRITC, Sigma-Aldrich) at 4 °C overnight and mounted with fluoroshield mounting medium. Images were captured using LSM 900/980 confocal microscope (Carl Zeiss) under airyscan mode for super high-resolution imaging. Airyscan processing was performed after images were captured by ZEN 3.3 software. For each sample, at least 100 cells were captured for membrane ruffle quantification.

### Immunofluorescent staining

Frozen tissue sections or HCC cells that were seeded on glass coverslips were washed with PBS and fixed using 4% formaldehyde at room temperature for 30 min. Samples were washed with 0.1% Triton-X-100 in PBS for 15 min and blocked in 5% FBS in PBST (0.1% Tween20 in PBS) at room temperature for 1 h. Blocking solutions were removed and samples were stained in primary antibody diluted in blocking solution at 4 °C overnight. Anti-GLUT1 antibody (ab15309, Abcam) anti-Na–K–ATPase antibody (ab76020, Abcam) or anti-pimonidazole mouse IgG1 monoclonal antibody (HP2-100 Kit, Hypoxyprobe™ Plus Kit) was used. Samples were washed with PBST and were incubated in secondary antibody (A27304, Goat anti-Rabbit IgG (H + L) Alexa Fluor 488, Invitrogen; A32733, Goat anti-Rabbit IgG (H + L) Alexa Fluor Plus 647, Invitrogen) diluted in blocking solution in the dark for 90 min. After removal of secondary antibody, samples were washed with PBST and mounted with fluoroshield mounting medium. Images were captured using LSM 900/980 confocal microscope (Carl Zeiss) under confocal or airyscan mode. Data were analyzed by ZEN 3.3 and ImageJ (64-bit Java 1.8.0_172) software^71,72^.

### Transmission electronic microscope

HCC cells were exposed to 21 and 1% O_2_ for 48 h. Cells were fixed with ice-cold 4% formalin at 4 °C overnight. Cells were scraped on ice and centrifuged at high speed. Cells were then fixed using 2.5% glutaraldehyde in cacodylate buffer (0.1 M sodium cacodylate–HCl, pH 7.4), rinsed with cacodylate buffer with 0.1 M sucrose, and fixed in 1% osmium tetroxide (OsO_4_). After fixation, cells were embedded into prewarmed 2% agar that was cut into 1 mm^3^ for dehydration and infiltration with epoxy resin. Cell cubes were embedded into fresh epoxy resin in plastic capsules and polymerized at 60 °C overnight. Images were captured by Philips CM100 TEM at 2950X.

### Transferrin uptake assay

The transferrin-uptake assay was performed as previously described^73^. HCC cells were exposed to 21 and 1% O_2_ for 48 h. Cells were serum-starved for 30 min and were harvested by trypsinization. Cells were resuspended in serum-free media and incubated with 50 µg/mL ice-cold transferrin, Alexa Fluor™ 633 Conjugate (T23362, Invitrogen) on ice for 10 min and at 37 °C for 10 min. Cells were washed with ice-cold serum-free media, PBS, and acidic buffer (0.1 M glycine, 150 mM NaCl, pH 3.0) and resuspended in ice-cold PBS for flow-cytometry analysis using BD LSRFortessa^TM^ flow cytometer (BD Biosciences). Data were analyzed by FlowJo v10.7 software (FlowJo, LLC).

### Fatty acid uptake assay

HCC cells were seeded onto a 6-well plate and exposed to 21 and 1% O_2_ for 48 h. Cells were serum-starved for 2 h and incubated with 2 µM BODIPY™ FL C12 (4,4-difluoro-5,7-dimethyl-4-bora-3a,4a-diaza-s-indacene-3-dodecanoic acid) (D3822, Invitrogen) diluted in serum‐free media for 30 min at 37 °C. After incubation, cells were washed with ice-cold PBS for three times and were resuspended in ice-cold PBS. Flow-cytometry analysis was performed by BD LSRFortessa^TM^ flow cytometer (BD Biosciences) and data were processed by FlowJo v10.7 software (FlowJo, LLC).

### Stable isotope labeling

^13^C,^15^N-DMEM were reconstituted from DMEM powder (D9800-26, US Biologics) with 4.5 g/L U^13^C-glucose (CLM-1396, Cambridge Isotope Laboratories), 1.6 g/L ^13^C,^15^N-amino acid mixture (CNLM-452, Cambridge Isotope Laboratories), 4 mM U^13^C–L-glutamine (CLM-1822, Cambridge Isotope Laboratories), 1 mM sodium pyruvate and 10% dialyzed FBS (26400044, Gibco). MHCC97L-EV, -HIF-1α-KO#58, and -EHD2-KO#4 cells were cultured with ^13^C,^15^N-DMEM for 8 cell doublings. An equal number of HCC subclones were cultured in 10% AA-^13^C,^15^N-DMEM (4.5 g/L U^13^C-glucose, 0.16 g/L ^13^C,^15^N-AAs, 0.2 mM U^13^C-Gln, and 5% dialyzed FBS) with unlabeled BSA (5% w/v) and were exposed to 21 and 1% O_2_ for 24 h. Intracellular metabolites were extracted, as we previously described^74^.

### Metabolite extraction

Cells were washed with 5% (w/v) mannitol (M4125, Sigma-Aldrich) and incubated in 100% methanol containing 10 μM internal standard (Human Metabolome Technologies, HMT). Cell suspensions were mixed with 0.5 mL of ultrapure water and centrifuged. Supernatants were added to ultracentrifugation columns (HMT) for high-speed centrifugation for 4 h at 4 °C. The elutes were collected for capillary electrophoresis time-of-flight mass spectrometry (CE-TOFMS) analysis (HMT). The absolute amounts of total and isotope-labeled metabolites were calculated according to standard curves. ^13^C,^15^N-labeled metabolites were defined as the metabolites with isotope labeling. The ^13^C,^15^N-labeled fraction of amino acid was relative to the total amount of amino acid.

### Animal experiments

BALB/cAnN-nude mice were used for orthotopic implantation and subcutaneous injection. Wild-type C57BL/6 N (*Ehd2*^+/+^) and C57BL/6 N *Ehd2* transgenic KO (*Ehd2*^−/−^) mice were used for HDTVi and DEN/CCl_4_ injection. In all animal experiments, male mice were used. Mice were housed with a dark/light cycle of 12 h, ambient temperature of 22 °C, and humidity of 30–70%.

For orthotopic implantation, 6–8-week-old nude mice were implanted with 1.5 × 10^6^ luciferase-labeled MHCC97L cells in 100% matrigel (354234, BD Biosciences). Mice were sacrificed 6 weeks after implantation. Mice were injected with 100 mg/kg D-luciferin (LUCK-1g, GoldBio). Bioluminescent imaging for mice and lung tissues was performed using PE-IVIS Spectrum In Vivo Imaging System (PerkinElmer). For subcutaneous injection, 2 × 10^6^ luciferase-labeled MHCC97L cells were resuspended in 50% matrigel in PBS. Cells were injected into flanks of nude mice. Three dimensions of the tumors were measured with caliper and values were inserted into the following formula for calculating tumor volume: tumor length x tumor width x tumor height x 0.52 mm^3^.

For HDTVi, 2–2.5 mL of genome-editing plasmids in saline were injected into 8–10 week-old mice (20–25 g) within 6–8 s through the tail vein as described^38^. Mice were injected with the CRISPR–Cas9 plasmids with sgRNAs targeting *Tp53*/*Keap1* and the Sleeping Beauty Transposon system over-expressing *c-Myc*, and the transposase in 10:5:1 ratio, as described^38^. Injected volume was equivalent to 10% of the weight of mice. For somatic deletion of *Ehd2*, sgRNAs targeting *Ehd2* were linked with the sgRNAs targeting *Tp53*. sgRNA sequence targeting *Ehd2* is provided in Supplementary Table 1. For DEN/CCl_4_ model, 14-day-old WT and *Ehd2*-KO mice (*Ehd2*^+/+^, *Ehd2*^−/−^) were intraperitoneally injected with a single dose of 5 mg/kg DEN (N0258, Sigma-Aldrich). About 8 weeks later, mice were injected with 0.5 mL/kg CCl_4_ (289116, Sigma-Aldrich) biweekly through intraperitoneal injection for 10 weeks. Mice were sacrificed 25 weeks after the last CCl_4_ administration.

All animal experiments and study protocols were approved by the Committee on the Use of Live Animals in Teaching and Research (CULATR) of the University of Hong Kong according to the Animals (Control of Experiments) Ordinance of Hong Kong. For subcutaneous tumor model, the tumor volumes did not exceed 10% of normal body weight or 1.3 cm in diameter. Mice were sacrificed if the percentage of body weight loss is greater than 20%. For orthotopic implantation and HDTVi model, mice were euthanasia if the percentage of body-weight gain is greater than 10% or weight loss is greater than 20%. The maximal tumor size/burden was permitted by CULATR of the University of Hong Kong. The maximal tumor size/burden was not exceeded in all experiments.

### Transgenic mice generation and genotyping

C57BL/6 N *Ehd2* transgenic KO mouse line was generated by Biocytogen, LLC (Beijing). EGE/CRISPR system was used for mouse-line generation. Two sgRNAs were designed to generate an ~1 kb chromosomal deletion (exon 3) at the *Ehd2* locus in the mouse genome. *Ehd2*-KO mice were bred in Minimal Disease Area of Laboratory Animal Unit under the guideline of the Committee on the Use of Live Animals for Teaching and Research of the University of Hong Kong. For genotyping, genomic DNA was extracted from ear-punched tissues. PCR was performed using AmpliTaq Gold polymerase (N8080246, Applied Biosystems^TM^). PCR product of wild type allele was 257 bp and the product of the KO allele was 536 bp. Primer sequences are provided in Supplementary Table 2.

### Immune-cell characterization

Tumor tissues were dissociated into single-cell suspensions by gentleMACS^TM^ dissociator (Miltenyl Biotec) in serum-free DMEM-F12 with DNase1 and Liberase, as described^75^. Red blood cells were lysed with ACK lysis buffer. After treatment with Fc Block^TM^ (553142, BD Biosciences), antibodies were added to cell suspensions for staining different cell-surface markers for 30 min at 4 °C. Antibodies were listed as follows: mouse CD3 (100205, Biolegend), mouse CD45 (103127; Biolegend), mouse CD4 (126619, Biolegend), mouse CD8 (126619, Biolegend), mouse CD11b (101205, Biolegend), mouse IAIE (107625, Biolegend), mouse F4/80 (123115, Biolegend), mouse Gr1 (108415, Biolegend), and mouse CD11c (117307, Biolegend). Cells were analyzed by BD LSRFortessa^TM^ flow cytometer (BD Biosciences) and data were processed by FlowJo v10.7 software (FlowJo, LLC). CD4^+^ T-cell population was defined as CD3^+^CD4^+^/CD45^+^%, CD8^+^ T-cell population was defined as CD3^+^CD8^+^/CD45^+^%, macrophage population was defined as F4/80^+^/CD45^+^%, MDSC population was defined as CD11b^+^Gr1^+^/CD45^+^%, and DC population was defined as CD11b^+^CD11c^+^/CD45^+^% or IAIE^+^/CD11c^+^%. The gating strategies for immune-cell population analysis were provided in Supplementary Fig. 20.

### Statistical analysis

Data were presented as mean ± standard deviation. All statistical analyses were performed by GraphPad Prism 7.0 or above software using two-tailed Student’s *t*-test, one-way ANOVA with Bonferroni correction, and two-way ANOVA with Bonferroni correction when appropriate. *P*-value < 0.05 was considered as statistically significant. The exact *P*-values were provided within figures.

### Reporting summary

Further information on research design is available in the Nature Research Reporting Summary linked to this article.

## Supplementary information

Supplementary Information

Reporting Summary

## Data Availability

The mass spectrometry data generated in this study are provided in the Source Data file. The EHD2 mRNA-expression values in human cancers were retrieved from the transcriptome-sequencing data available at the Cancer Genome Atlas (TCGA) (cBioportal for Cancer Genomics: http://www.cbioportal.org/). Megalin (LRP2) and cubilin (CUBN) expressions in human tissues and cancers were retrieved from The Human Protein Atlas (LRP2, CUBN). All the other data are available within the article and its Supplementary Information. Source data are provided with this paper.

## Source data

Source Data
