# Supplementary material for: Hypoxia-induced macropinocytosis represents a metabolic route for liver cancer
Source: Nat Commun. 2022 Feb 17;13:954. doi: 10.1038/s41467-022-28618-9 (PMC8854584; doi:10.1038/s41467-022-28618-9)
Supplement: Supplementary file 1 — Supplementary Information [file 41467_2022_28618_MOESM1_ESM.pdf]

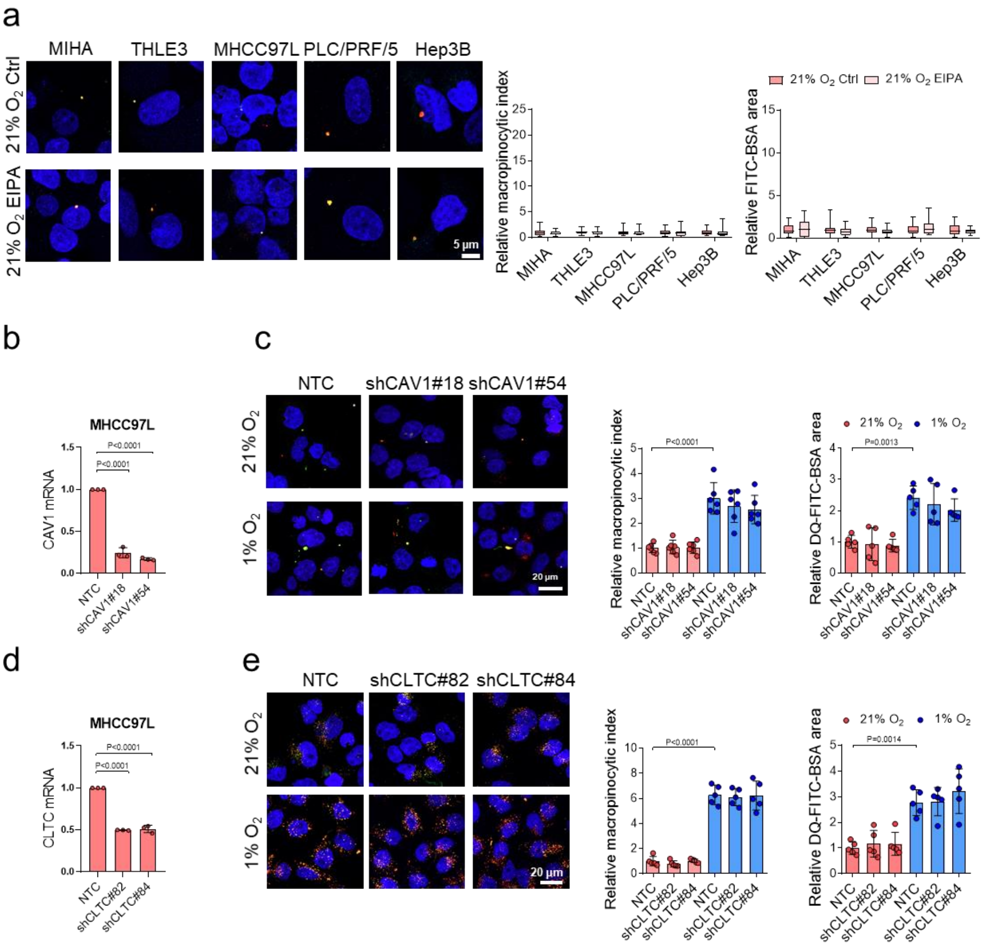

Supplementary Figure 1 Hypoxia induces dextran uptake and BSA degradation through macropinocytosis in HCC

a. MIHA, THLE3, MHCC97L, PLC/PRF/5 and Hep3B exposed to 21% O_2 were treated with 50 μM EIPA or vehicle control (Ctrl). Confocal images show macropinosome/dextran (red) and constitutively fluorescent BSA (green). Macropinocytic indexes were calculated based on the intensities of red signals, constitutively fluorescent BSA particle areas were calculated from green signals. Values were normalized to 21% O_2 Ctrl. **b.** CAV1 mRNA expressions of MHCC97L-NTC, -shCAV1#18, -shCAV1#54 cells exposed to 21% O_2. **c.** Confocal images show dextran uptake/macropinosomes (red, n=6 independent imaging analysis) and FITC-BSA degradation (green, n=5 independent imaging analysis) of MHCC97L-NTC, -shCAV1#18, -shCAV1#54 cells exposed to 21% and 1% O_2. **d.** CLTC mRNA expressions of MHCC97L-NTC, -shCLTC#82, -shCLTC#84 cells exposed to 21% O_2. **e.** Confocal images show dextran uptake/macropinosomes (red) and FITC-BSA degradation (green) of MHCC97L-NTC, -shCLTC#82, -shCLTC#84 cells exposed to 21% and 1% O_2. n=5 independent imaging analysis. **a, b, d:** Results were from 3 independent experiments. **b, d:**

mRNA expressions were normalized to 18S. **c, e:** Macropinocytic indexes and relative DQ-FITC-BSA areas were normalized to 21% O_2 NTC. Error bars indicate mean ± SD. Box-and-whisker: center line, median; box limits, 25th to 75th percentiles; whiskers, Min to Max. **a, c, e:** Two-way ANOVA with Bonferroni correction. **b, d:** One-way ANOVA with Bonferroni correction. Source data are provided as a Source Data file.

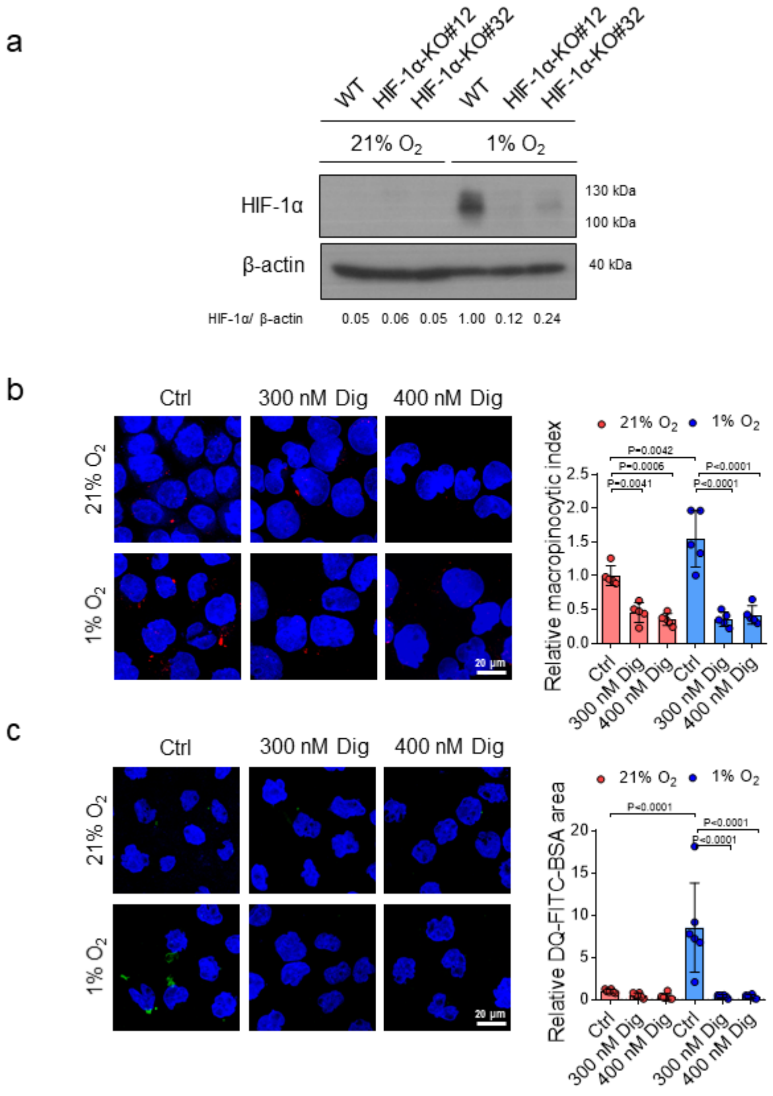

Supplementary Figure 2 HIF-KO/inhibition blocks hypoxia-induced macropinocytosis in HCC cells

a. Western blots show HIF-1α protein expressions in MHCC97L-wild type (WT), -HIF-1α-KO#12, -HIF-1α-KO#32 cells exposed to 21% and 1% O_2. Band intensities were normalized to 1% MHCC97L-WT. **b.** Confocal images demonstrate dextran uptake/macropinosomes (red) of MHCC97L cells treated with 300 nM digoxin (Dig), 400 nM Dig, or vehicle control (Ctrl) exposed to 21% and 1% O_2. Macropinocytic indexes were normalized to 21% O_2 Ctrl. n=5 independent imaging analysis. **c.** Confocal images demonstrate FITC-BSA degradation (green) of MHCC97L cells treated with Dig or Ctrl exposed to 21% and 1% O_2. DQ-FITC-BSA particle areas were normalized to 21% O_2 Ctrl. n=6 independent imaging analysis. Error bars indicate mean ± SD. Two-way ANOVA with Bonferroni correction. Source data are provided as a Source Data file.

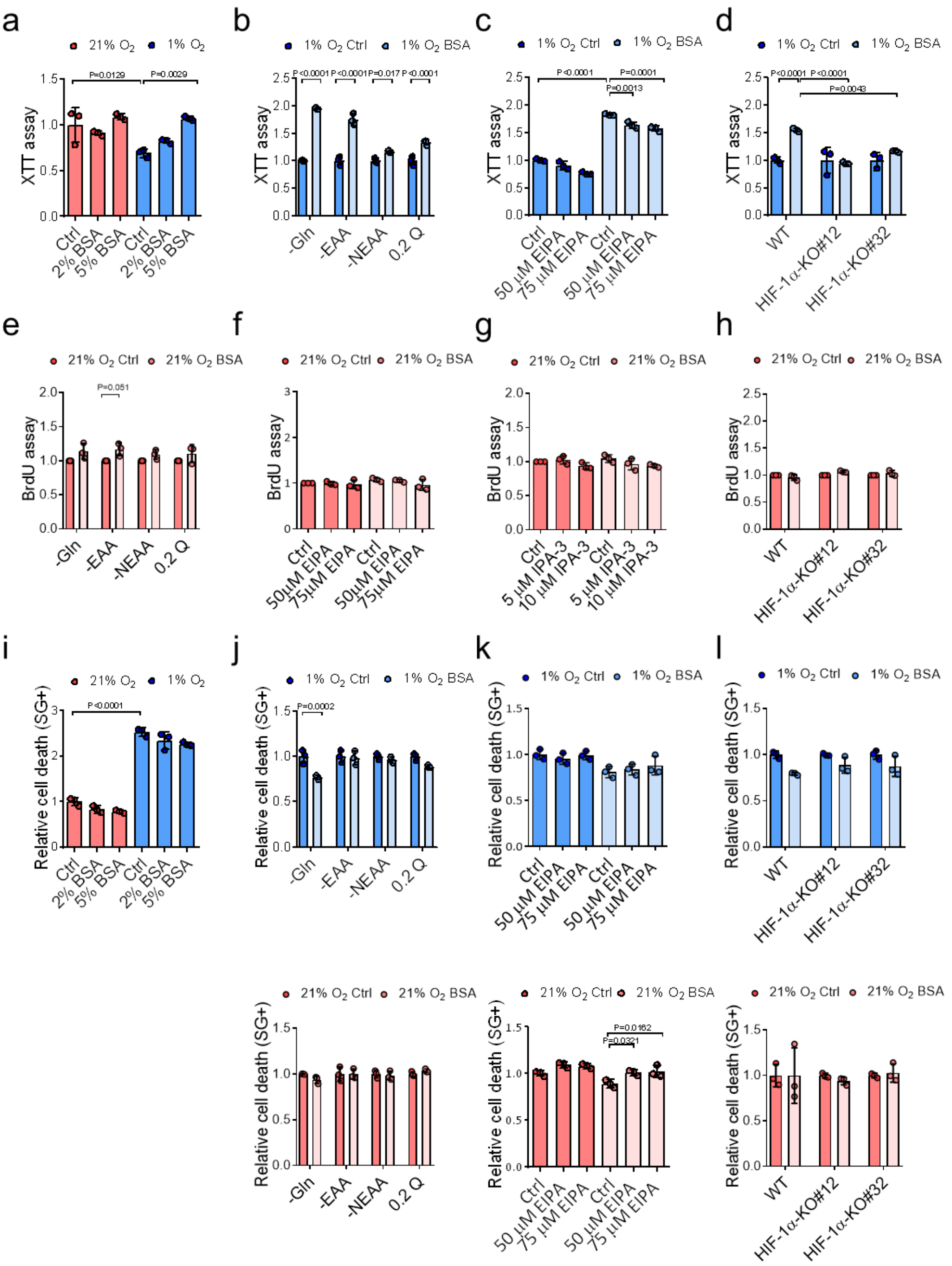

Supplementary Figure 3 Protein scavenging promotes hypoxic HCC cell growth but not cell death

a. Cell proliferation of MHCC97L cells cultured in 0.2 Q DMEM media supplemented with BSA or vehicle control. Values were normalized to 21% O_2 Ctrl. **b, e.** Cell proliferation of MHCC97L cells cultured in -Gln, -EAA, -NEAA, or 0.2 Q DMEM treated with 5% BSA or vehicle control (PBS). **c, f.** Cell proliferation of MHCC97L cells cultured in 10% AA-DMEM containing 5% BSA. Cells were treated with EIPA or vehicle control (DMSO). **d, h.** Cell proliferation of MHCC97L-WT, -HIF-1α-KO#12, and -HIF-1α-KO#32 cultured in 10% AA-DMEM treated with 5% BSA or vehicle control (PBS). **g.** Cell proliferation of MHCC97L cells cultured in 10% AA-DMEM treated with IPA-3 or vehicle control (DMSO). Cells were

supplemented with 5% BSA or vehicle control (PBS). **i.** Percentage of dead cells of MHCC97L cells cultured in 0.2 Q DMEM media supplemented with BSA (2%, 5% w/v) or vehicle control (PBS) in 21% and 1% O_2. **j.** Percentage of dead cells of MHCC97L cells cultured in -Gln, -EAA, -NEAA, or 0.2 Q DMEM in 21% and 1% O_2 treated with 5% BSA or vehicle control (PBS). **k.** Percentage of dead cells of MHCC97L cells cultured in 10% AA-DMEM containing 5% BSA. Cells were treated with EIPA or vehicle control (DMSO). **l.** Percentage of dead cells of MHCC97L-WT, -HIF-1α-KO#12, and -HIF-1α-KO#32 cultured in 10% AA-DMEM treated with 5% BSA or vehicle control (PBS) under 21% and 1% O_2. **a-d:** Cell proliferations were measured using XTT assays. **b-d:** Values were normalized to 1% O_2 Ctrl. **e-h:** Corresponds to **Figure 2b-e** respectively. Cell proliferations were measured by BrdU labelling in 21% O_2. Values were normalized to 21% O_2 Ctrl. **i-l:** Dead cell populations were calculated as SYTOX Green positive populations (SG+). Values were normalized to 21% and 1% O_2 Ctrl, respectively. **a-d:** Results were representative for 3 independent experiments. **e-h:** Results were from 3 independent experiments. **i-l:** n=3 independent samples. Error bars indicate mean ± SD. Two-way ANOVA with Bonferroni correction. Source data are provided as a Source Data file.

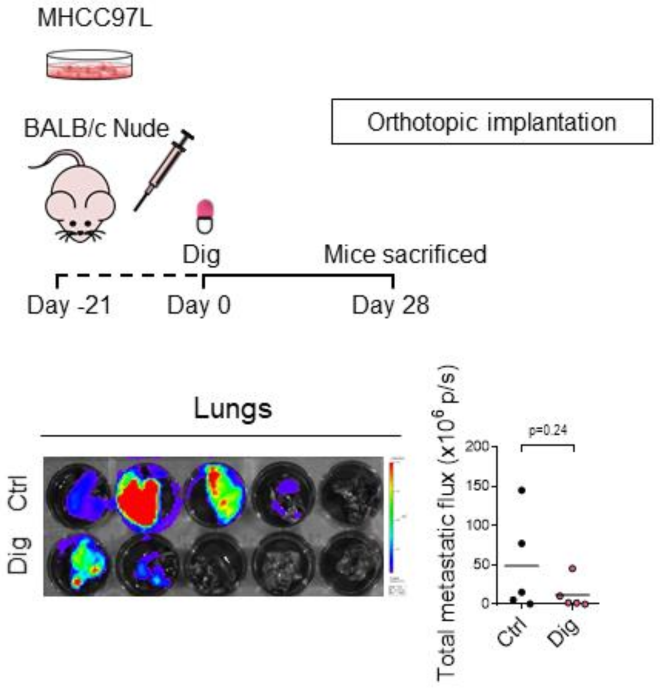

Supplementary Figure 4 Digoxin inhibits HCC lung metastasis *in vivo*

6-8 weeks old male nude mice were orthotopically implanted with luciferase-labeled MHCC97L cells and were treated with digoxin (Dig) (1.2 mg/kg/day, i.p.) or vehicle control (Ctrl, saline) for 28 days. Bioluminescent image of lung metastases was shown, and luciferase intensities were quantified. n=5 independent animals. The number of mice was represented as the number of dots. Center line, mean. Two tailed Student's t-test. Source data are provided as a Source Data file.

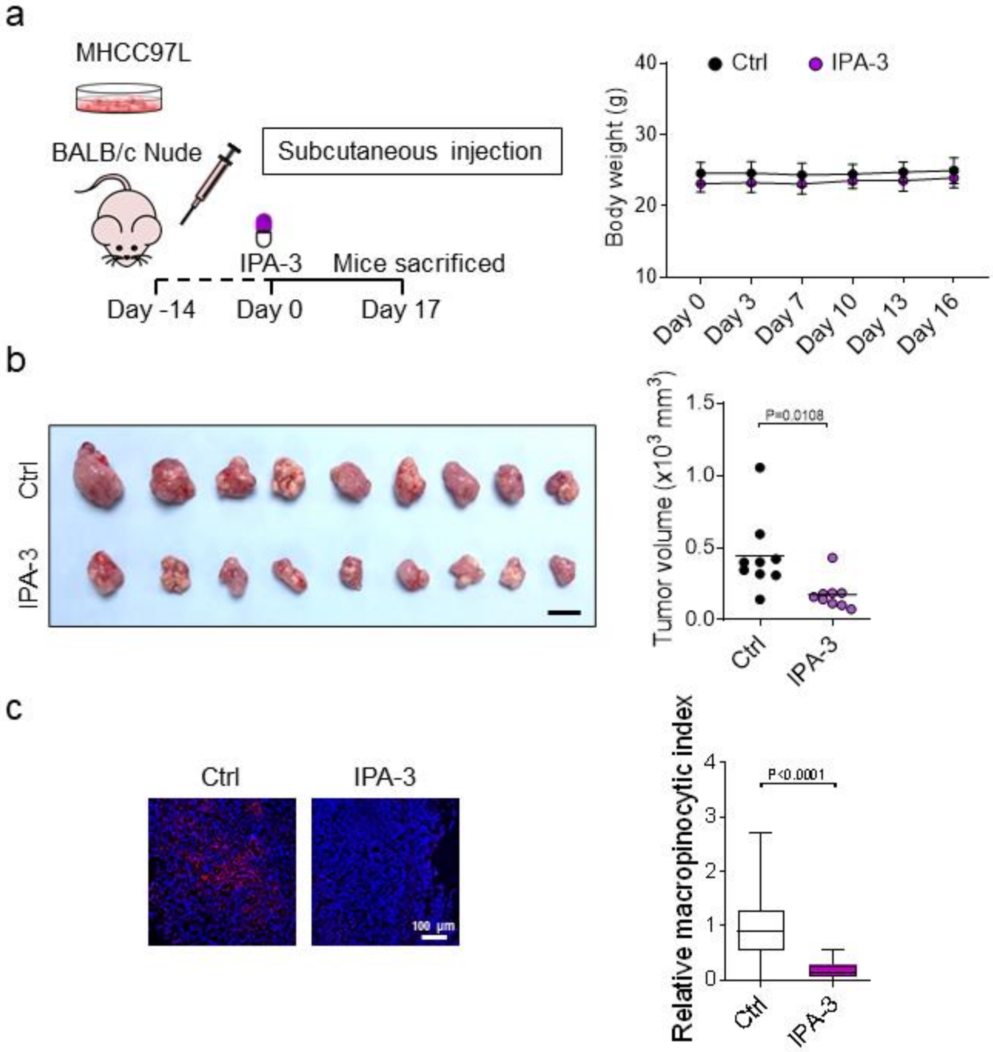

Supplementary Figure 5 IPA-3 inhibits HCC growth *in vivo* through blockade of macropinocytosis

a. Left: 6-8 weeks old male nude mice were subcutaneously injected with 2 x 10^6 luciferase-labeled MHCC97L cells. Mice received IPA-3 (2 mg/kg/biweekly, i.p.) or vehicle control (Ctrl, 10% DMSO in β-captisol) for 17 days. Right: Body weights of mice during IPA-3 treatment. n=5 independent animals. **b.** Image of HCC xenografts and tumor volumes. n=9 independent tumors. **c.** Confocal images demonstrate *ex vivo* dextran uptake/macropinosomes (red) and nuclei (DAPI, blue) of HCC tissues. Macropinocytic indexes were normalized to Ctrl. Scale bar, 1 cm. Error bars indicate mean ± SD. n=9 independent samples. Scatter plot: center line, mean. Box-and-whisker: center line, median; box limits, 25th to 75th percentiles; whiskers, Min to Max. Two tailed Student's t-test. Source data are provided as a Source Data file.

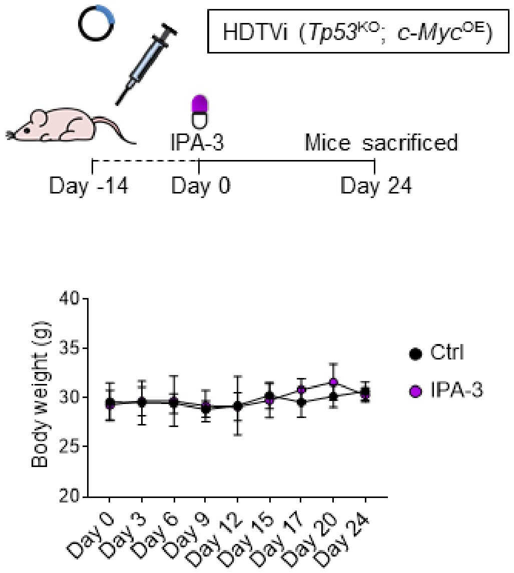

Supplementary Figure 6 Body weights of mice receiving IPA-3

HDTVi was performed in 8-10 weeks old male C57BL/6N mice to induce *Tp53*^{KO}; *c-Myc*^{OE} mouse HCC. Mice were received IPA-3 (2 mg/kg/biweekly, i.p.) or vehicle control (Ctrl, 10% DMSO in β-captisol) for 24 days. Body weights of mice during IPA-3 treatment were shown. n=6 mice. Error bars indicate mean ± SD. Source data are provided as a Source Data file.

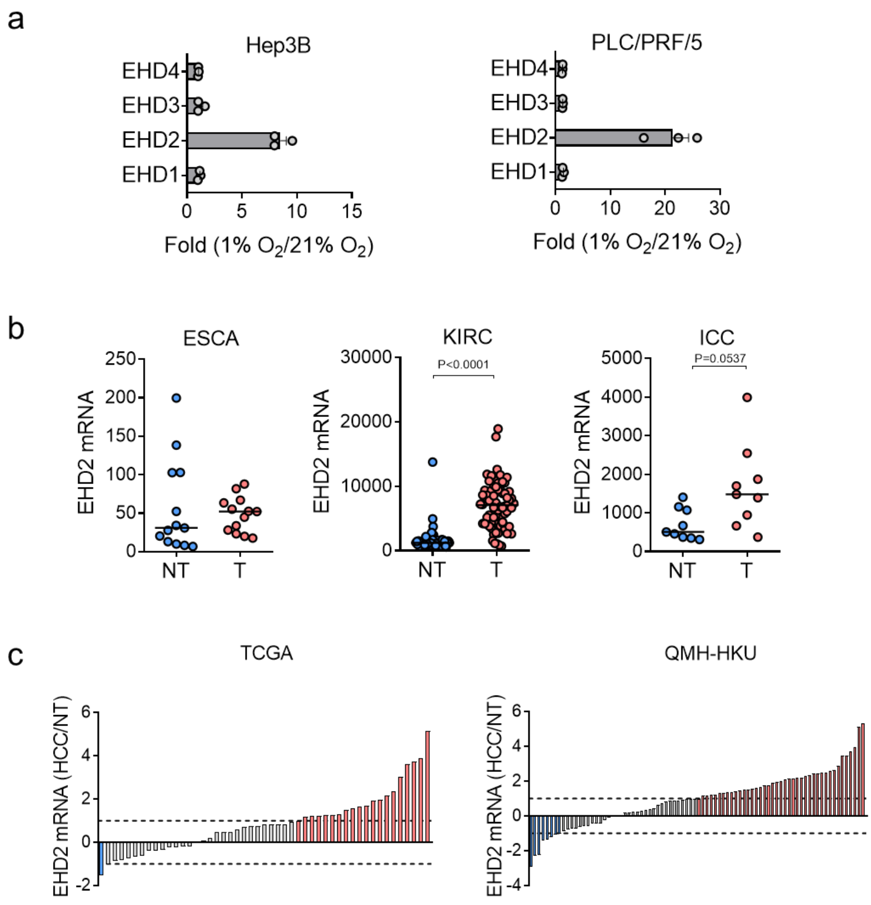

Supplementary Figure 7 Overexpression of EHD2 in HCC and different cancer types

a. RT-qPCR data demonstrate the mRNA expressions of EHD family (EHD1-EHD4) in Hep3B and PLC/PRF/5 cells exposed to 21% and 1% O_2. Values were normalized to 21% O_2. mRNA expressions were normalized to 18S. Results were from 3 biologically independent experiments. **b.** Transcriptome sequencing data from the TCGA show EHD2 mRNA expressions in tumor (T) and corresponding NT tissues from 13 cases of esophageal carcinoma (ESCA), 72 cases of kidney renal clear cell carcinoma (KIRC) and 9 cases of intrahepatic cholangiocarcinoma (ICC) respectively. **c.** Left: Waterfall plot demonstrate EHD2 mRNA expressions in 49 HCC cases from the TCGA database. Right: Waterfall plot show EHD2 mRNA expressions in 82 HCC cases from QMH-HKU cohort. Values were normalized to NT. Two tailed Student's t-test. Source data are provided as a Source Data file.

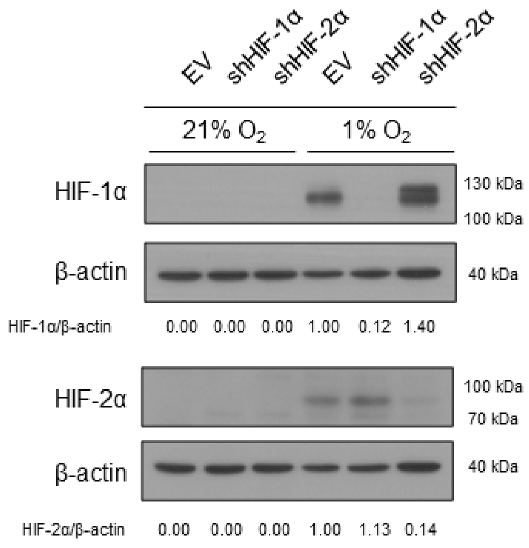

Supplementary Figure 8 HIF-1α/HIF-2α expressions in MHCC97L-shHIF-1α/-shHIF-2α cells

Western blots show HIF-1α and HIF-2α protein expressions in MHCC97L-empty vector (EV), -shHIF-1α and -shHIF-2α cells exposed to 21% and 1% O_2. Band intensities were normalized to 1% MHCC97L-EV. Source data are provided as a Source Data file.

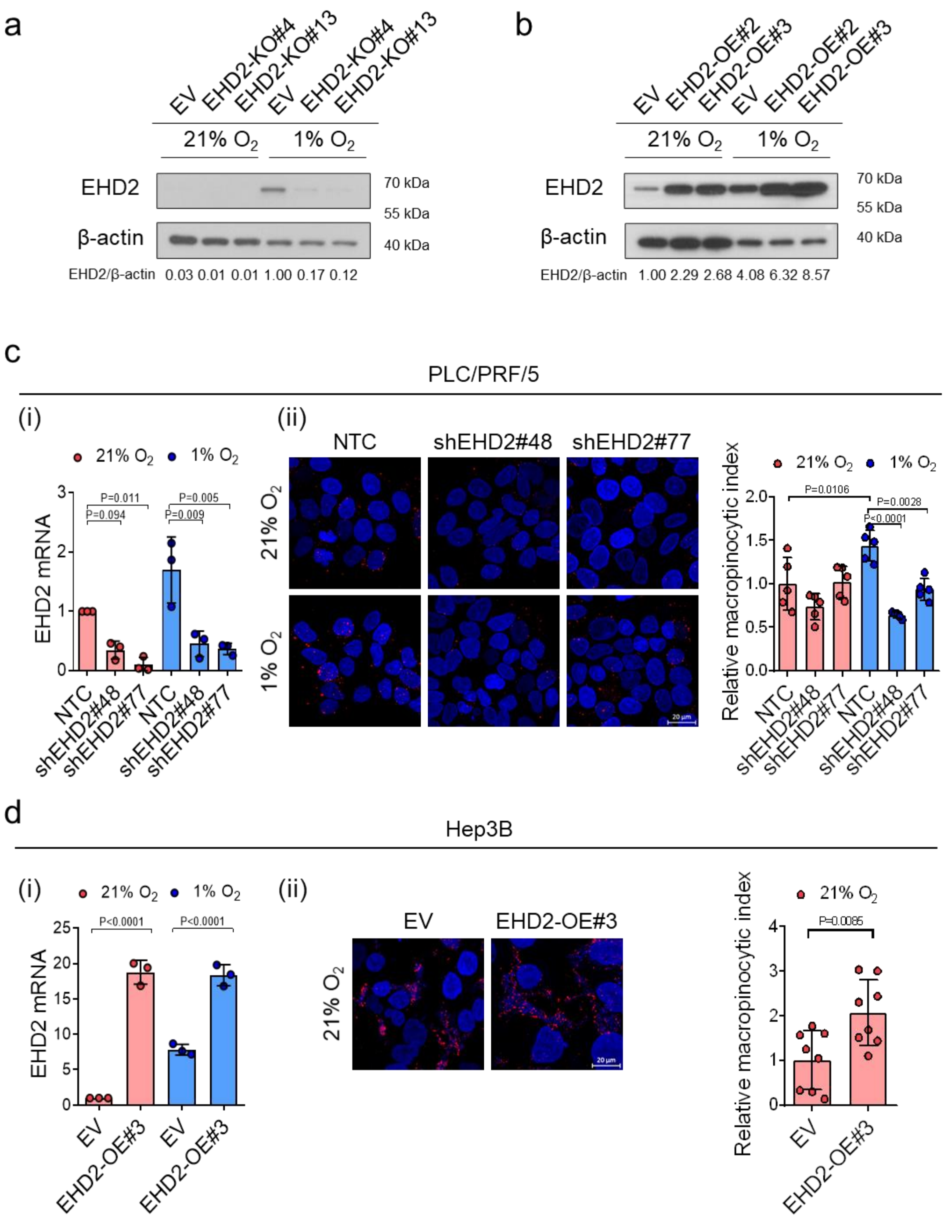

Supplementary Figure 9 EHD2 promotes hypoxia-induced macropinocytosis

a. Western blots show EHD2 protein expressions in MHCC97L-EV, -EHD2-KO#4, -EHD2-KO#13 cells exposed to 21% and 1% O_2. Band intensities were normalized to 1% O_2 EV. The experiments were repeated for 3 times with similar results.**b.** Western blots show EHD2 protein expressions in MHCC97L-EV, -EHD2-OE#2, -EHD2-OE#3 cells exposed to 21% and 1% O_2. Band intensities were normalized to 21% O_2 EV. **c. (i)** EHD2 mRNA expressions of PLC/PRF/5-NTC, -shEHD2#48, -shEHD2#77 cells exposed to 21% and 1% O_2. Results were from 3 independent experiments. **(ii)** Confocal images demonstrate dextran uptake/macropinosomes (red) and nuclei (DAPI, blue) of PLC-NTC, -shEHD2#48, -shEHD2#77 subclones. Macropinocytic indexes were normalized to 21% O_2 NTC. n=8

independent imaging analysis. **d. (i)** EHD2 mRNA expressions of Hep3B-EV and -EHD2-OE#3 subclones exposed to 21% and 1% O_2. Results were from 3 independent experiments. **(ii)** Confocal images show dextran uptake/macropinosomes (red) and nuclei (DAPI, blue) of Hep3B-EV, -EHD2-OE#3 exposed to 21% O_2. Macropinocytic indexes were normalized to EV. n=8 independent imaging analysis. Error bars indicate mean ± SD. **a, b:** The results were representative for 3 independent experiments with similar results. **c, d (i):** Two-way ANOVA with Bonferroni correction. **d (ii):** Two tailed Student's t-test. Source data are provided as a Source Data file.

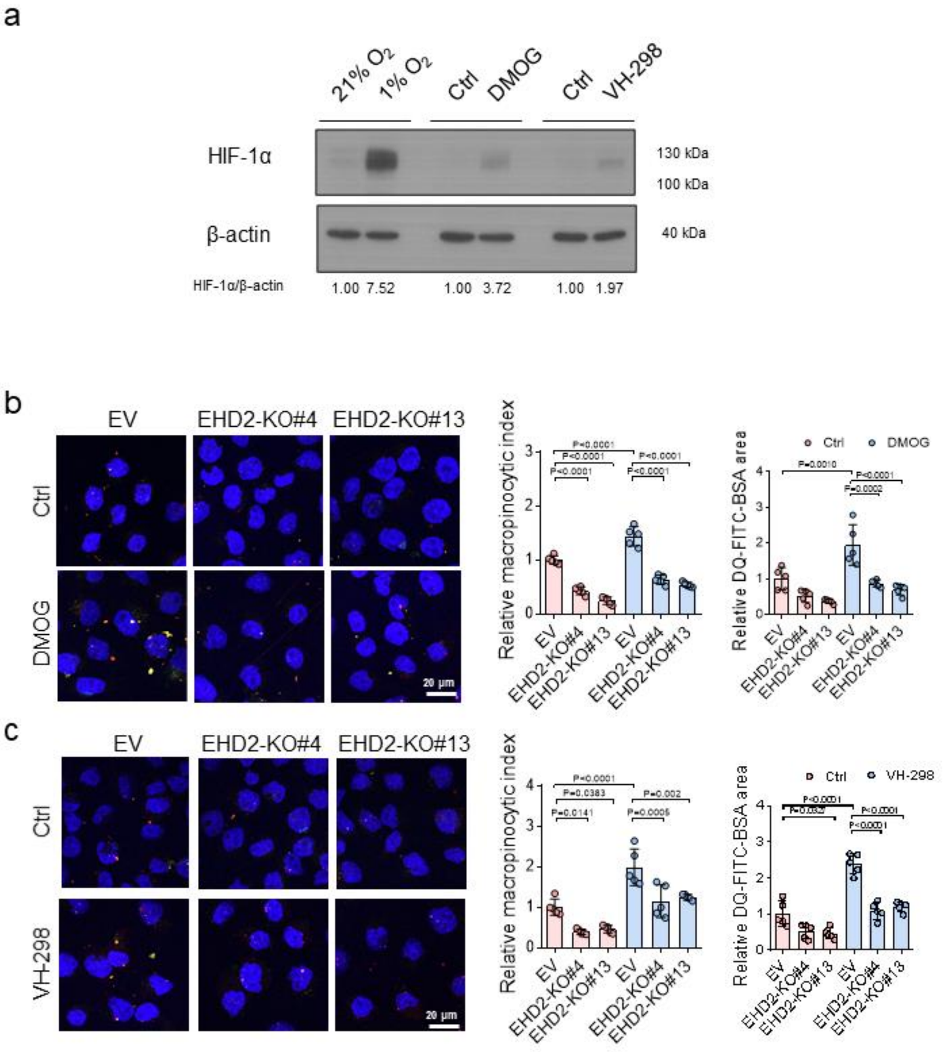

Supplementary Figure 10 HIF activation induces macropinocytosis

a. Western blots show HIF-1α protein expressions in MHCC97L cells exposed to 21% or 1% O_2, MHCC97L cells treated with 500 µM DMOG or vehicle control exposed to 21% O_2, and MHCC97L cells treated with 200 µM VH-298 or vehicle control exposed to 21% O_2. Band intensities were normalized to 21% O_2 or Ctrl. The results were representative for 2 independent experiments with similar results. **b.** MHCC97L-EV, -EHD2-KO#4, -EHD2-KO#13 cells exposed to 21% O_2 were treated with 500 µM DMOG or vehicle control (Ctrl, H_2O). Confocal images show dextran uptake/macropinosomes (red) and FITC-BSA degradation (green). n=5 independent imaging analysis. **c.** MHCC97L-EV, -EHD2-KO#4, -EHD2-KO#13 cells exposed to 21% O_2 were treated with 50 µM VH-298 or vehicle control (Ctrl, DMSO). Confocal images show dextran uptake/macropinosomes (red) and FITC-BSA degradation (green). n=5 independent imaging analysis. Macropinocytic indexes and DQ-FITC-BSA particle areas were normalized to 21% O_2 EV Ctrl. Error bars indicate mean ± SD. Two-way ANOVA with Bonferroni correction. Source data are provided as a Source Data file.

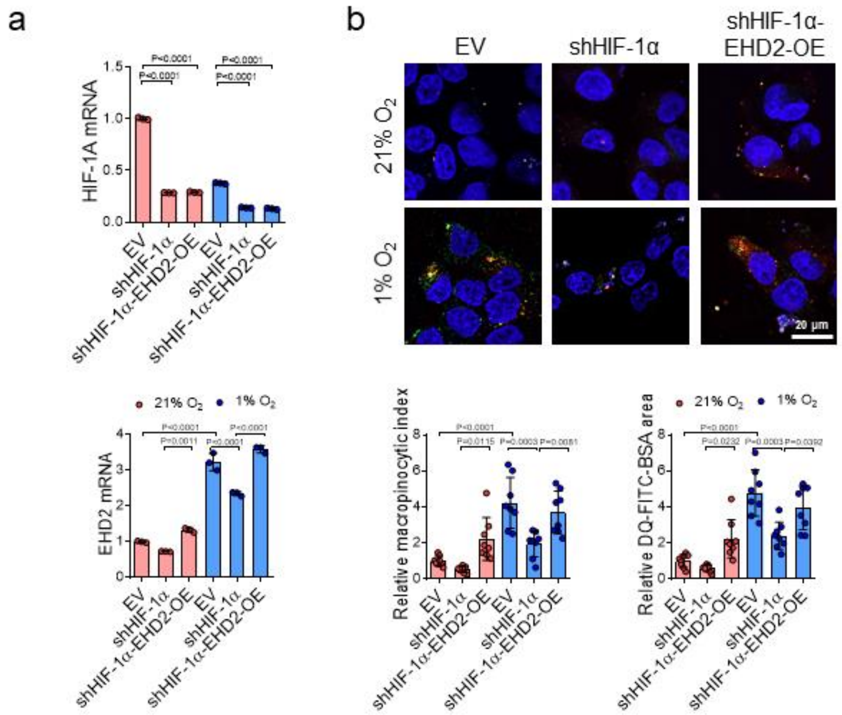

Supplementary Figure 11 HIF activation induces macropinocytosis through induction of EHD2

a. MHCC97L-EV, MHCC97L-shHIF-1α, MHCC97L-shHIF-1α-EHD2-OE cells were exposed to 21% and 1% O_2. HIF-1A mRNA and EHD2 mRNA were measured. mRNA expressions were normalized to 18S. Results were representative for 3 independent experiments with similar results. **b.** Confocal images show dextran uptake/macropinosomes (red) and FITC-BSA degradation (green) of MHCC97L-EV, -shHIF-1α, -shHIF-1α-EHD2-OE cells exposed to 21% and 1% O_2. Macropinocytic indexes and DQ-FITC-BSA particle areas were normalized to 21% O_2 EV. n=8 independent imaging analysis. Error bars indicate mean ± SD. Two-way ANOVA with Bonferroni correction. Source data are provided as a Source Data file.

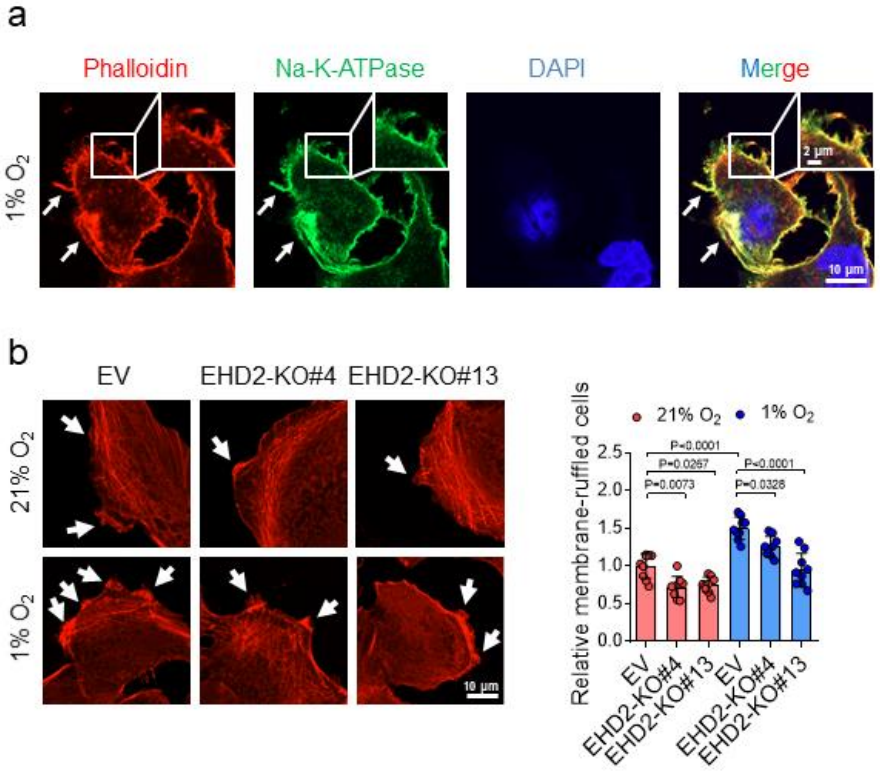

Supplementary Figure 12 Hypoxia induces membrane ruffles formation through upregulation of EHD2

a. Super high-resolution images show actin (phalloidin, red), plasma membrane (Na-K-ATPase, green) and nuclei (DAPI, blue) of MHCC97L-EV cells exposed to 1% O_2. Arrows indicate membrane ruffles on the plasma membrane. Inserts indicate macropinocytic cup structure. Results were representative for 2 independent imaging analysis. **b**. Super high-resolution images show actin filaments (phalloidin, red) in MHCC97L-EV, -EHD2-KO#4, -EHD2-KO#13 cells exposed to 21% and 1% O_2. Arrows indicate the membrane ruffles. Percentage of cells with membrane ruffles were normalized to 21% O_2 EV. n=9 independent imaging analysis. Error bars indicate mean ± SD. Two-way ANOVA with Bonferroni correction. Source data are provided as a Source Data file.

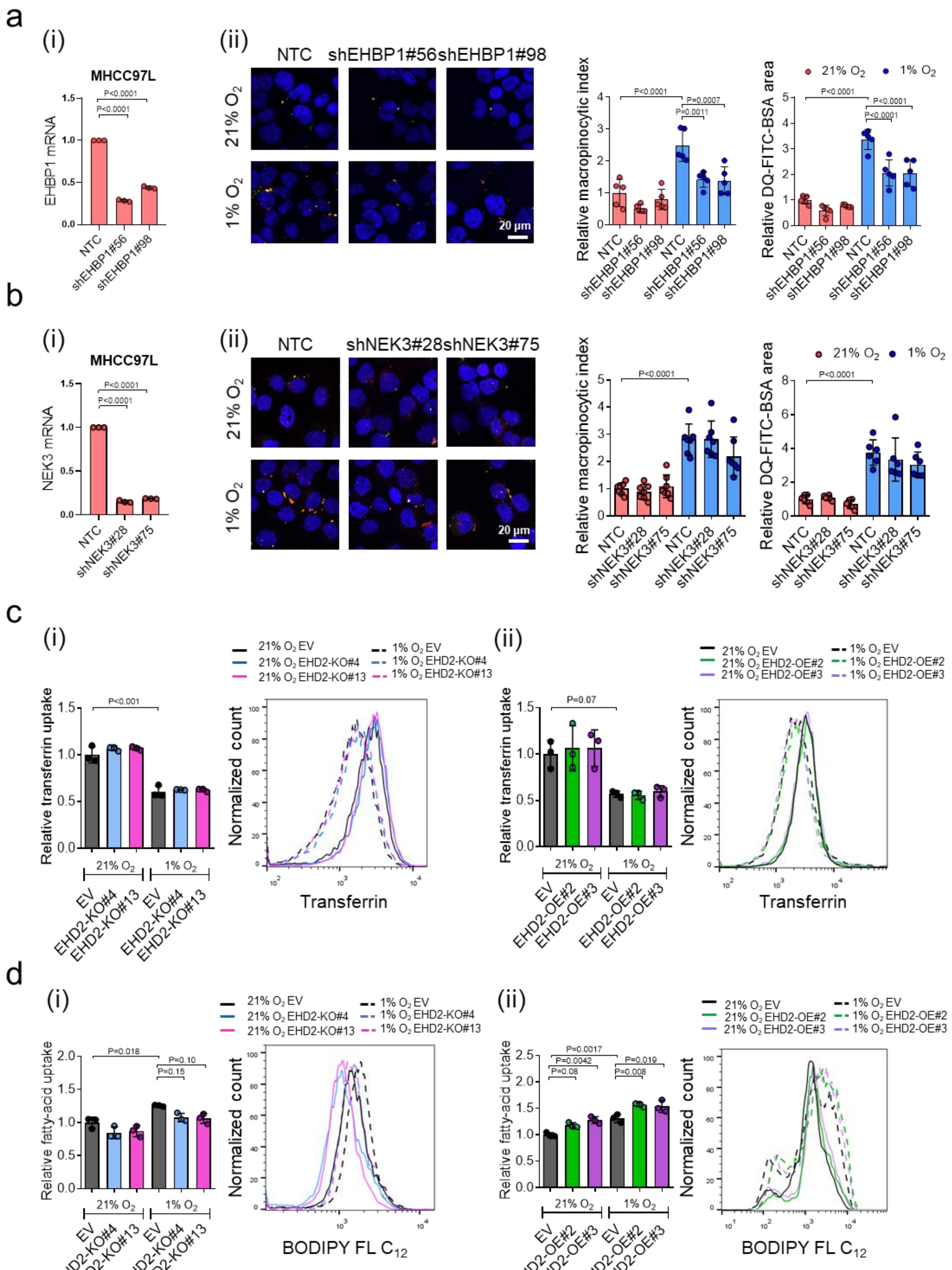

Supplementary Figure 13 EHD2 interacts with plasma membrane to regulate multiple endocytic pathways

a. (i) EHBP1 mRNA expressions of MHCC97L-NTC, -shEHBP1#56, -shEHBP1#98 cells exposed to 21% O_2. Results were from 3 independent experiments. **(ii)** Confocal images show dextran uptake/macropinosomes (red) and FITC-BSA degradation (green) of MHCC97L-NTC, -shEHBP1#56, -shEHBP1#98 cells exposed to 21% and 1% O_2. n=5 independent

imaging analysis. **b.** **(i)** NEK3 mRNA expressions of MHCC97L-NTC, -shNEK3#28, -shNEK3#75 cells exposed to 21% O_2. Results were from 3 independent experiments. **(ii)** Confocal images show dextran uptake/macropinosomes (red) and FITC-BSA degradation (green) of MHCC97L-NTC, -shNEK3#28, -shNEK3#75 cells exposed to 21% and 1% O_2. n=7 independent imaging analysis. **c.** **(i)** Transferrin uptake of MHCC97L-EV, -EHD2-KO#4, -EHD2-KO#13 cells exposed to 21% and 1% O_2. **(ii)** Transferrin uptake of MHCC97L-EV, -EHD2-OE#2, -EHD2-OE#3 cells exposed to 21% and 1% O_2. n=3 independent samples. **d.** **(i)** Fatty acid uptake indicated by BODIPY FL C_{12} of MHCC97L-EV, -EHD2-KO#4, -EHD2-KO#13 cells exposed to 21% and 1% O_2. **(ii)** Fatty acid uptake of MHCC97L-EV, -EHD2-OE#2, -EHD2-OE#3 cells exposed to 21% and 1% O_2. n=3 independent samples. **a-b:** All mRNA values were normalized to 18S. Macropinocytic indexes and DQ-FITC-BSA particle area were normalized to 21% O_2 NTC. **c-d:** Values were normalized to 21% O_2 EV. Error bars indicate mean ± SD. One-way ANOVA with Bonferroni correction. **a (ii), b (ii), c-d:** Two-way ANOVA with Bonferroni correction. Source data are provided as a Source Data file.

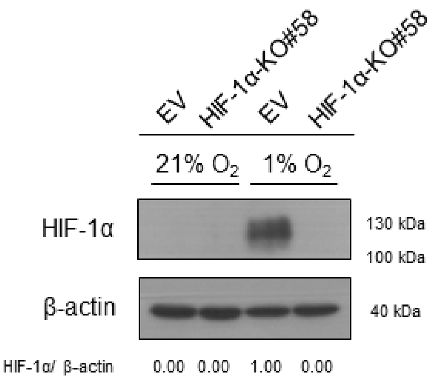

Supplementary Figure 14 HIF-1α expression in MHCC97L-HIF-1α-KO cells

Western blots show HIF-1α protein expressions in MHCC97L-EV and -HIF-1α-KO#58 exposed to 21% and 1% O_2. Band intensities were normalized to 1% O_2 EV. Results were representative for 3 independent experiments with similar results. Source data are provided as a Source Data file.

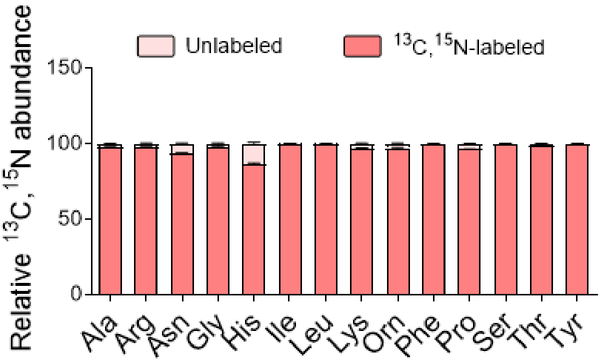

Supplementary Figure 15 Relative abundance of intracellular ^{13}C,^{15}N-amino acids in MHCC97L-EV cells

Abundance of ^{13}C,^{15}N-labeled and unlabeled amino acids in MHCC97L-EV cells cultured in ^{13}C,^{15}N-DMEM after 8 cell doublings under 21% O_2 condition. Results were from 3 independent mass spectrometry analysis. Values were normalized to total amino acid abundance. Error bars indicate mean ± SD. Ala, alanine; Arg, arginine; Asn, asparagine; Gly, glycine; His, histidine; Ile, isoleucine; Leu, leucine; Lys, lysine; Orn, ornithine; Phe, phenylalanine; Pro, proline; Ser, serine; Thr, threonine; Tyr, tyrosine. Source data are provided as a Source Data file.

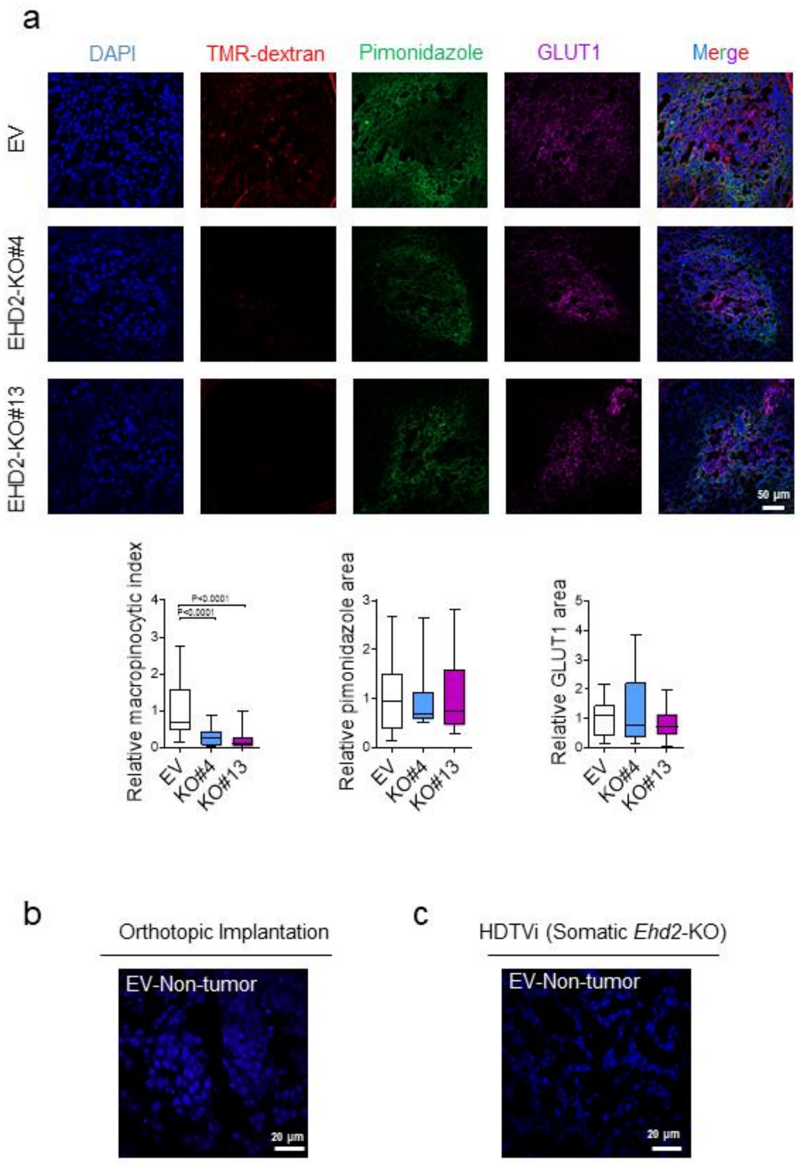

Supplementary Figure 16 *In vivo* dextran uptake of HCC and liver tissues

a. Confocal images show *in vivo* dextran uptake/macropinosomes (red), pimonidazole (green), GLUT1 (purple) and nuclei (DAPI, blue) of HCC tumors harvested from MHCC97L-EV, -EHD2-KO#4, -EHD2-KO#13 orthotopic implantation model. **b**. Confocal images show *in vivo* dextran uptake/macropinosomes (red) and nuclei (DAPI, blue) of non-tumorous liver tissue harvested from MHCC97L-EV orthotopic implantation model. **c**. Confocal images show *in vivo* dextran uptake/macropinosomes (red) and nuclei (DAPI, blue) of non-tumorous liver tissue harvested from EV of HDTVi model. **a.** n=5 mice for each experimental group. Macropinocytic indexes, pimonidazole and GLUT1 signal areas were normalized to EV. **b-c.** Images were representative for 5 samples. Box-and-whisker: center line, median; box limits, 25th to 75th percentiles; whiskers, Min to Max. Two-way ANOVA with Bonferroni correction. Source data are provided as a Source Data file.

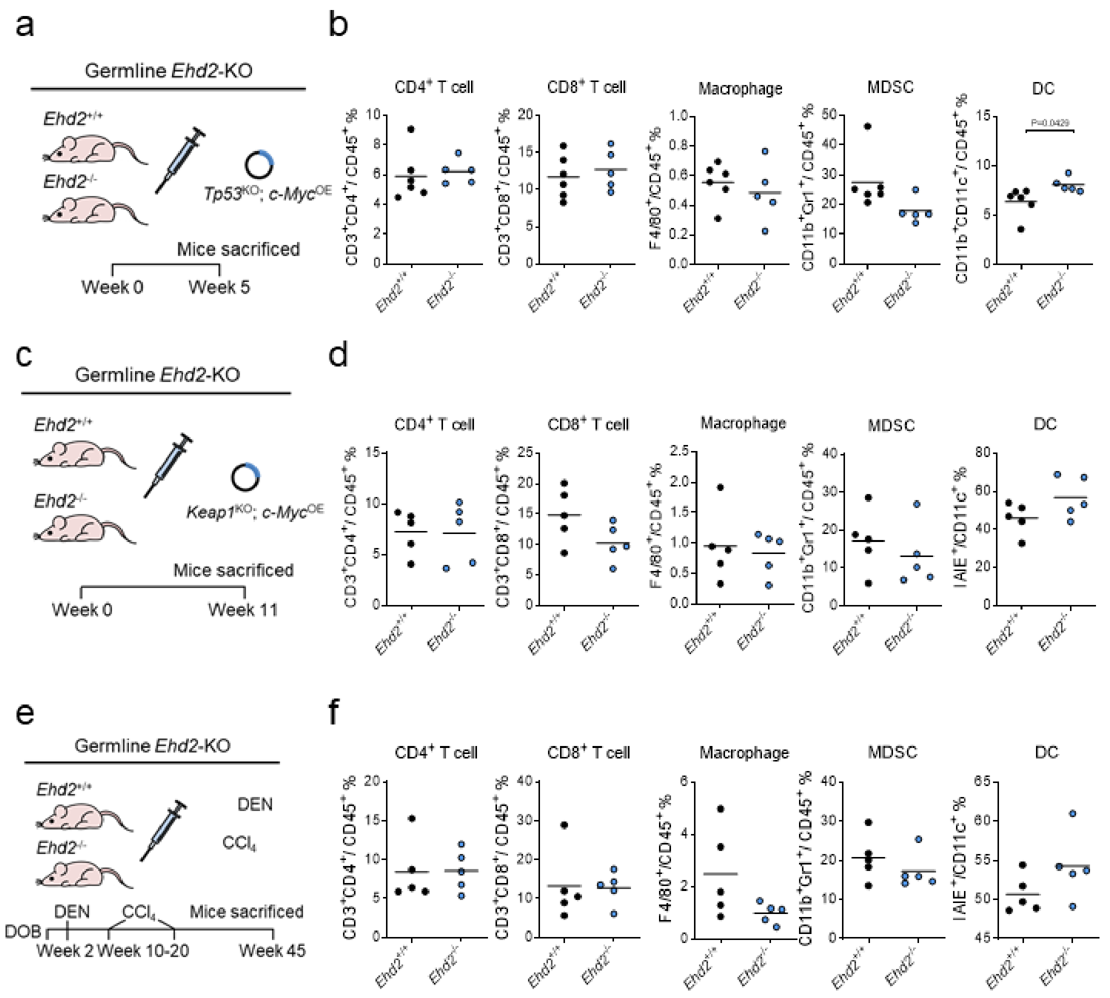

Supplementary Figure 17 KO of *Ehd2* does not affect immune cell populations in HCC tissues

a. HDTVi was performed in 8-10 weeks old male C57BL/6N EHD2 transgenic mice (*Ehd2*$^{+/+}$, *Ehd2*$^{-/-}$) to generate *Tp53*^{KO}; *c-Myc*^{OE} HCC. Mice were sacrificed 5 weeks after HDTVi. (Corresponds to **Figure 7c**). **b.** Immune cell populations in *Tp53*^{KO}; *c-Myc*^{OE} HCC tissues from *Ehd2*$^{+/+}$/*Ehd2*$^{-/-}$ mice (Corresponds to **Figure 7c**). **c.** HDTVi was performed in 8-10 weeks old male *Ehd2*$^{+/+}$ and *Ehd2*$^{-/-}$ mice to induce *Keap1*^{KO}; *c-Myc*^{OE} mouse HCCs. Mice were sacrificed 11 weeks after HDTVi (Corresponds to **Figure 7d**). **d.** Immune cell populations in *Keap1*^{KO}; *c-Myc*^{OE} HCC tissues from *Ehd2*$^{+/+}$/*Ehd2*$^{-/-}$ mice (corresponds to **Figure 7d**). **e**. Hepatocarcinogens, DEN and CCl_4, were injected into male *Ehd2*$^{+/+}$ and *Ehd2*$^{-/-}$ mice. Mice were sacrificed 45 weeks after birth (Corresponds to **Figure 7e**). **f.** Immune cell populations in DEN/CCl_4 HCC tissues from *Ehd2*$^{+/+}$/*Ehd2*$^{-/-}$ mice (corresponds to **Figure 7e**). The number of mice was represented as the number of dots. Center line, mean. Two tailed Student's t-test. Source data are provided as a Source Data file.

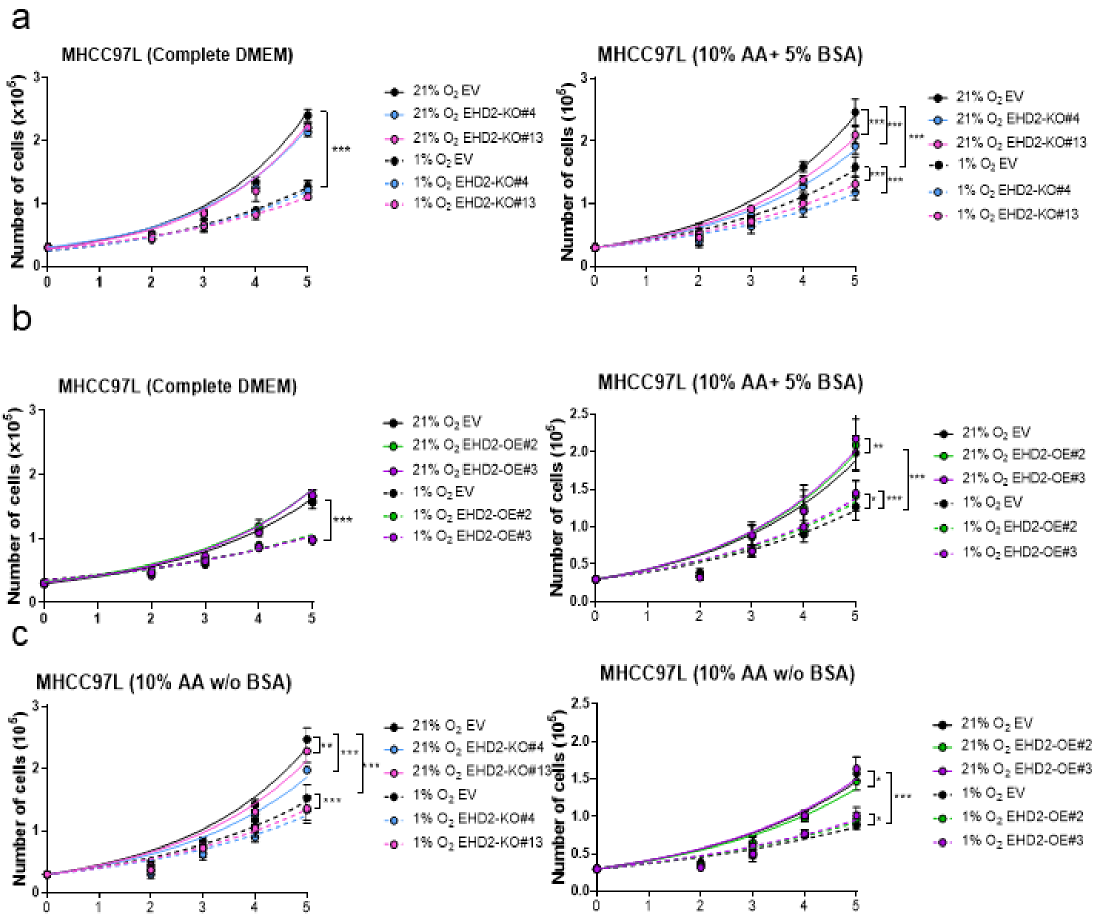

Supplementary Figure 18 Cell proliferation of MHCC97L-EHD2-KO/OE subclones *in vitro*

a. Left: Number of cells of MHCC97L-EV, -EHD2-KO#4, -EHD2-KO#13 cells exposed to 21% and 1% O_2. Right: Number of cells of MHCC97L-EV, -EHD2-KO#4, -EHD2-KO#13 cells cultured in 10% AA-DMEM with 5% BSA supplement exposed to 21% and 1% O_2. **b.** Left: Number of cells of MHCC97L-EV, -EHD2-OE#2, -EHD2-OE#3 cells exposed to 21% and 1% O_2. Right: Number of cells of MHCC97L-EV, -EHD2-OE#2, -EHD2-OE#3 cells cultured in 10% AA-DMEM with 5% BSA exposed to 21% and 1% O_2. **c.** Left: Number of cells of MHCC97L-EV, -EHD2-KO#4, -EHD2-KO#13 cells cultured in 10% AA-DMEM exposed to 21% and 1% O_2. Right: Number of cells of MHCC97L-EV, -EHD2-OE#2, -EHD2-OE#3 cells cultured in 10% AA-DMEM exposed to 21% and 1% O_2. Results were from 3 independent experiments. Error bars indicate mean ± SD. $*P < 0.05$, $**P < 0.01$, $***P < 0.001$. Two-way ANOVA with Bonferroni correction. Source data are provided as a Source Data file.

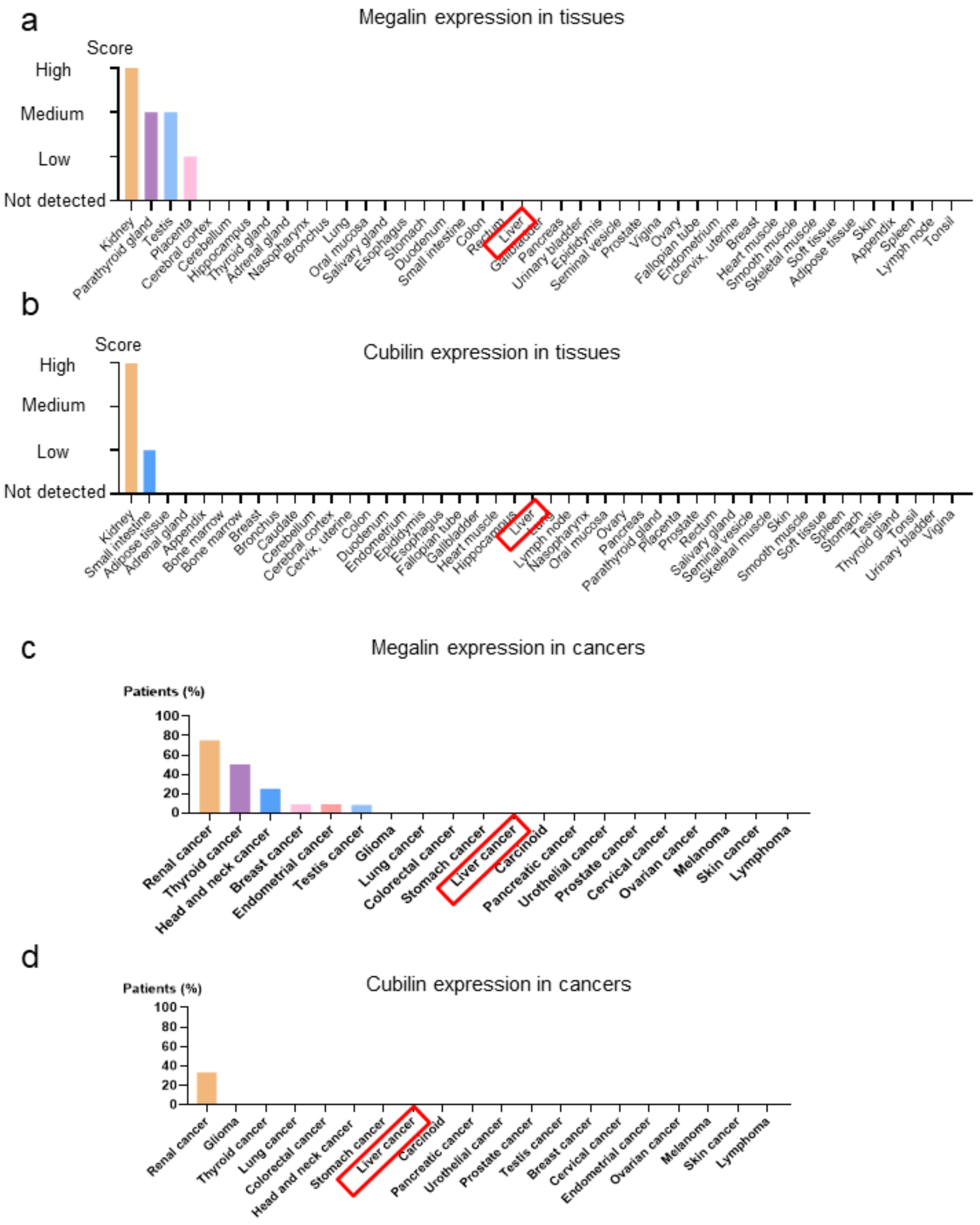

Supplementary Figure 19 Megalin and Cubilin expressions in tissues and cancers

a. Megalin expressions in human tissues retrieved from immunohistochemistry data available from the Tissue Atlas of The Human Protein Atlas. **b.** Cubilin expressions in human tissues retrieved from immunohistochemistry data available from the Tissue Atlas of The Human Protein Atlas. **c.** Megalin expressions in cancers retrieved from immunohistochemistry data available from the Pathology Atlas of The Human Protein Atlas. **d.** Cubilin expressions in cancers retrieved from immunohistochemistry data available from the Pathology Atlas of The Human Protein Atlas.

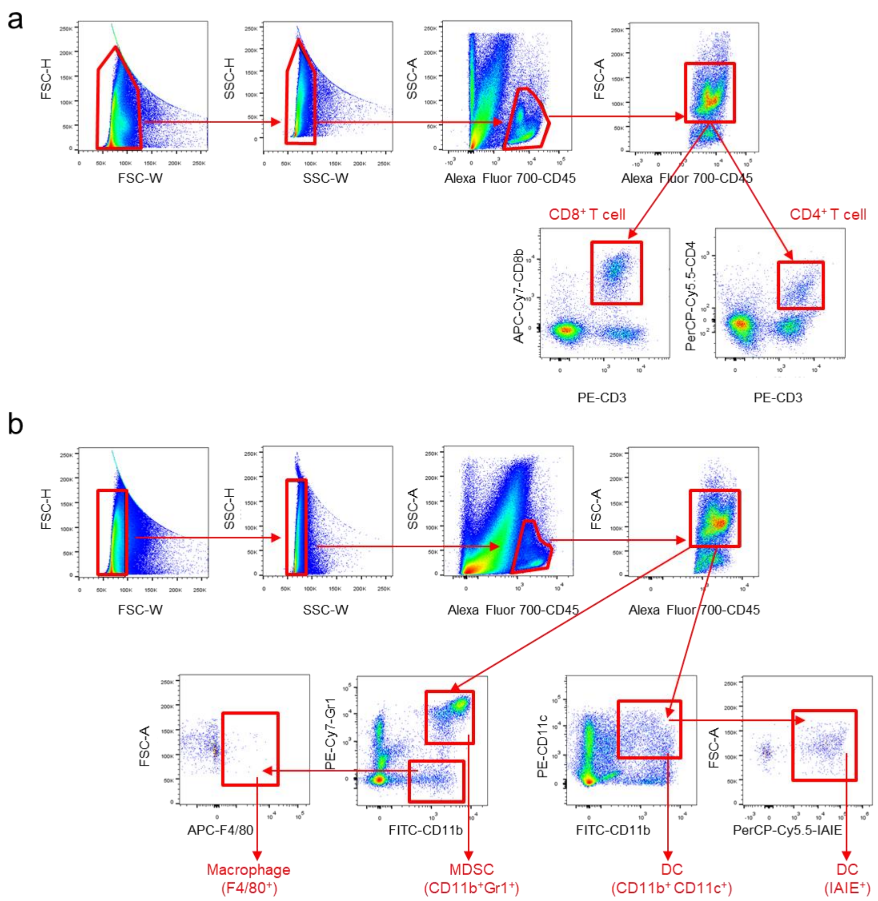

Supplementary Figure 20 Gating strategies for immune cell populations

a. Gating strategies for $CD4^+$ and $CD8^+$ T cells. $CD4^+$ T-cell population was defined as $CD3^+CD4^+/CD45^+$%, $CD8^+$ T-cell population was defined as $CD3^+CD8^+/CD45^+$%. **b.** Gating strategies for macrophage, myeloid-derived suppressor cell (MDSC), and dendritic cell (DC). Macrophage population was defined as $F4/80^+/CD45^+$%, MDSC population was defined as $CD11b^+Gr1^+/CD45^+$%, DC population was defined as $CD11b^+CD11c^+/CD45^+$% or $IAIE^+/CD11c^+$%.

Supplementary Table 1 shRNA and sgRNA target sequences

shRNA/ sgRNA	Target Sequence
**Human sh*EHD2*#48**	GCCCGTCATCTTTGCGAAGAT
**Human sh*EHD2*#77**	GCGAGTTCACGCTTACATCAT
**Human sg*EHD2*-Cas9-#4**	TCGTGCGGTACAGCTCCTT
**Human sg*EHD2*-Cas9-#13**	ATGTTCAGCTGGCTGAAGC
**Human sg*HIF-1A*-Cas9-#58**	AACCATAACAAAACCATCCA
**Human sg*EHD2*-dCas9-#2**	GAGCAGCCCAACGCTGGAG
**Human sg*EHD2*-dCas9-#3**	TTAAGAAGAGAGACAGACT
**Human sh*HIF-1A***	GTTACGTTCCTTCGATCAG
**Human sh*CAV1*#18**	GACCCACTCTTTGAAGCTGTT
**Human sh*CAV1*#54**	AGAGCTTCCTGATTGAGATTC
**Human sh*CLTC*#82**	GCCCAAATGTTAGTTCAAGAT
**Human sh*CLTC*#84**	GCCAATGTGATCTGGAACTTA
**Human sh*EHBP1*#56**	CCGAAGAAGAAGATGAGCATT
**Human sh*EHBP1*#98**	CATAGGAATTTCCCGATTATT
**Human sh*NEK3*#28**	CCCAATTTCCTCAACCATAAA
**Human sh*NEK3*#75**	CCTAGTCAAGCAGATGTTTAA
**Mouse sg*Ehd2*-Cas9**	GCCACGAAGCAGTCGGTGGT

Supplementary Table 2 Primer sequences

Primer	Sequence
**Human *EHD2***	Forward: CTGCTGCGAGTTCACGCTTA
	Reverse: ACGGGCAGTTTGAGGATCAG
**Human *EHD1***	Forward: ACAACCGCAAGCTCTTTGAG
	Reverse: TGATGTAGGCGTGAACCTTG
**Human *EHD3***	Forward: ACCGCATCATTCTGCTCTTC
	Reverse: TCAGCTTTGTTCAGCACCAC
**Human *EHD4***	Forward: TGAAGAGCATCAGCGTCATC
	Reverse: TTGTGAGCGTCAAAGAGCAG
**Human *EHD2* (ChIP)**	Forward: TCAAGCAATCTTCCCACC
	Reverse: AACACGGGAATCACAACA
**Human *HIF-1A***	Forward: TGAGCTTGCTCATCAGTTGC
	Reverse: CCAGAAGTTTCCTCACACGC
**Human *CAV1***	Forward: CATCTGGGGCATTTACTTCG
	Reverse: TGGAATAGACACGGCTGATG
**Human *CLTC***	Forward: TGCCCTATTTCATCCAGGTC
	Reverse: GGGGCTGACCATAAACAATG
**Human *EHBP1***	Forward: TATGCAAGCCCTATGCCAAC
	Reverse: CATCTGTGGCTTTTCCTTCC
**Human *NEK3***	Forward: TACCCAAATGTGCCTTGGAG
	Reverse: ATCGGATTGGAGAGAAGACG
**Human *18S***	Forward: GAGGATGAGGTGGAACGTGT
	Reverse: AGAAGTGACGCAGCCCTCTA
**Mouse *Ehd2*$^{+/+}$ (WT)**	Forward: CTACTTCTTTCCCTTCCTGCGCCTG
	Reverse: CCTGGGATATGGGGAGACAGAAGGT
**Mouse *Ehd2*$^{-/-}$ (KO)**	Forward: CTACTTCTTTCCCTTCCTGCGCCTG
	Reverse: CTTCTTCACCTCCTGTGGAAGCTGG
