# Supplementary material for: Hypoxia-induced macropinocytosis represents a metabolic route for liver cancer
Source: Nat Commun. 2022 Feb 17;13:954. doi: 10.1038/s41467-022-28618-9 (PMC8854584; doi:10.1038/s41467-022-28618-9)
Supplement: Supplementary file 2 — Reporting Summary [file 41467_2022_28618_MOESM2_ESM.pdf]

nature portfolio

Corresponding author(s): Carmen Chak-Lui Wong

Last updated by author(s): 2021-12-23

Reporting Summary

Nature Portfolio wishes to improve the reproducibility of the work that we publish. This form provides structure for consistency and transparency in reporting. For further information on Nature Portfolio policies, see our Editorial Policies and the Editorial Policy Checklist.

Statistics

For all statistical analyses, confirm that the following items are present in the figure legend, table legend, main text, or Methods section.

n/a	Confirmed	
☐	☒	The exact sample size (*n*) for each experimental group/condition, given as a discrete number and unit of measurement
☐	☒	A statement on whether measurements were taken from distinct samples or whether the same sample was measured repeatedly
☐	☒	The statistical test(s) used AND whether they are one- or two-sided<br>*Only common tests should be described solely by name; describe more complex techniques in the Methods section.*
☒	☐	A description of all covariates tested
☐	☒	A description of any assumptions or corrections, such as tests of normality and adjustment for multiple comparisons
☐	☒	A full description of the statistical parameters including central tendency (e.g. means) or other basic estimates (e.g. regression coefficient) AND variation (e.g. standard deviation) or associated estimates of uncertainty (e.g. confidence intervals)
☐	☒	For null hypothesis testing, the test statistic (e.g. *F*, *t*, *r*) with confidence intervals, effect sizes, degrees of freedom and *P* value noted<br>*Give P values as exact values whenever suitable.*
☒	☐	For Bayesian analysis, information on the choice of priors and Markov chain Monte Carlo settings
☒	☐	For hierarchical and complex designs, identification of the appropriate level for tests and full reporting of outcomes
☒	☐	Estimates of effect sizes (e.g. Cohen's *d*, Pearson's *r*), indicating how they were calculated

Our web collection on statistics for biologists contains articles on many of the points above.

Software and code

Policy information about availability of computer code

Data collection	Flow cytometry data was collected by BD LSRFortessaTM flow cytometer (BD Biosciences) ;<br>Imaging data was collected by Zen 3.3 (Carl Zeiss);<br>The number of cells was determined by TC20TM Automated Cell Counter (Bio-Rad);<br>The metabolomics assay was performed by Human Metabolome Technologies (Tokyo, Japan);<br>The luciferase image of mouse xegnograft was collected by Xenogen IVIS 100 Imaging System (Caliper, Hopkinton, MA, USA)
Data analysis	Flow cytometry data was analyzed using FlowJo (v10.7);<br>Imaging data was collected by Zen 3.3 (Carl Zeiss) and ImageJ (64-bit Java 1.8.0_172) ;

For manuscripts utilizing custom algorithms or software that are central to the research but not yet described in published literature, software must be made available to editors and reviewers. We strongly encourage code deposition in a community repository (e.g. GitHub). See the Nature Portfolio guidelines for submitting code & software for further information.

Data

Policy information about availability of data

All manuscripts must include a data availability statement. This statement should provide the following information, where applicable:

- Accession codes, unique identifiers, or web links for publicly available datasets
- A description of any restrictions on data availability
- For clinical datasets or third party data, please ensure that the statement adheres to our policy

The mass spectrometry data generated in this study are provided in the Source Data file. The database used in this study is TCGA (cBioPortal) [https://www.cbioportal.org/] and the Human Protein Atlas (https://www.proteinatlas.org/ENSG00000081479-LRP2; https://www.proteinatlas.org/ENSG00000107611-

CUBN).

Field-specific reporting

Please select the one below that is the best fit for your research. If you are not sure, read the appropriate sections before making your selection.

☒ Life sciences ☐ Behavioural & social sciences ☐ Ecological, evolutionary & environmental sciences

For a reference copy of the document with all sections, see nature.com/documents/nr-reporting-summary-flat.pdf

Life sciences study design

All studies must disclose on these points even when the disclosure is negative.

Sample size	No statistical methods were used to predetermine sample sizes and no sample size calculations were performed. Sample sizes were chosen based on the adequacies to provide statistically significant differences between experimental groups using similar experimental conditions across biological replicates.
Data exclusions	No data were excluded from the analyses except for data generated from experimental failures caused by technical errors such as equipment failure, expired/ spoiled reagents, sample contaminations, etc.
Replication	Experiment findings were based on 2 or 3 independent biological replicates with similar experimental conditions as mentioned in the figure legends and/ or main text unless specified otherwise.
Randomization	For animal experiments, tumour-bearing mice were randomly divided into different experimental groups prior to treatment to ensure tumour sizes were distributed evenly. For cell experiments, cells were randomly assigned to treatment or control group.
Blinding	Investigators were not blinded to group allocations during data collection as individual group or mice received different treatments. Analyses in the animal experiments as the results reported were based on measurements acquired.

Reporting for specific materials, systems and methods

We require information from authors about some types of materials, experimental systems and methods used in many studies. Here, indicate whether each material, system or method listed is relevant to your study. If you are not sure if a list item applies to your research, read the appropriate section before selecting a response.

Materials & experimental systems

n/a	Involved in the study	
☐	☒	Antibodies
☐	☒	Eukaryotic cell lines
☒	☐	Palaeontology and archaeology
☐	☒	Animals and other organisms
☐	☒	Human research participants
☒	☐	Clinical data
☒	☐	Dual use research of concern

Methods

n/a	Involved in the study	
☒	☐	ChIP-seq
☐	☒	Flow cytometry
☒	☐	MRI-based neuroimaging

Antibodies

Antibodies used

ChIP antibodies:
HIF-1α (anti-mouse, Abcam, ab1,1:300); HIF-1β (anti-rabbit, Abcam, ab2, 1:300); IgG control (anti-rabbit, Santa Cruz Biotechnology, sc-2027, 1:300); IgG control (anti-mouse, Santa Cruz Biotechnology, sc-2762, 1:300).

Western blotting antibodies:
HIF-1α (anti-rabbit, Cell Signaling Technologies, #3716, 1:1000); HIF-2α (anti-rabbit, Abcam, ab199, 1:500) ; EHD2 (anti-rabbit, Abcam, ab154784, 1:1000); EHD2 (anti-rabbit, Invitrogen, PA5-49403, 1:1000); β-actin (anti-mouse, Sigma-Aldrich, A5316, 1:2500).

Immunofluorescence antibodies:
GLUT1 (anti-rabbit, Abcam, ab15309, 1:100); Na-K-ATPase (anti-rabbit, Abcam, ab76020, 1:100); FITC conjugated to anti-pimonidazole mouse IgG1 monoclonal antibody (FITC-MAb1) (anti-mouse, Hypoxyprobe, HP2-100Kit, 1:100); Goat-anti-Rabbit IgG (H+L), Alexa Fluor488 (Invitrogen, A27034, 1:600), Goat anti-Rabbit IgG (H+L) Highly Cross-Adsorbed Secondary Antibody, Alexa Fluor Plus 647 (Invitrogen, A32733, 1:600).

Flow antibodies:
CD3 antibody (anti-mouse, Biolegend, 100205, 1:100); CD45 antibody (anti-mouse, Biolegend, 103127, 1:100); CD4 antibody (anti-mouse, Biolegend, 100429, 1:100); CD8 antibody (anti-mouse, Biolegend, 126619, 1:100); CD11b antibody (anti-mouse, Biolegend, 101205, 1:100); IAIE antibody (anti-mouse, Biolegend, 107625, 1:100); F4/80 antibody (anti-mouse, Biolegend, 123115, 1:100); Gr1 antibody (anti-mouse, Biolegend, 108415, 1:100); CD11c antibody (anti-mouse, Biolegend, 117307, 1:100).

Validation	Validation statements for the species and application of all antibodies used in this study were taken from the respective datasheet and/or manufacturer's website.<br>HIF-1α (anti-mouse, Abcam, ab1) https://www.abcam.com/hif-1-alpha-antibody-h1alpha67-ab1.html<br>HIF-1β (anti-rabbit, Abcam, ab2) https://www.abcam.com/hif1-beta-antibody-chip-grade-ab2.html<br>IgG (anti-rabbit, Santa Cruz Biotechnology, sc-2027) https://datasheets.scbt.com/sc-2027.pdf<br>IgG control (anti-mouse, Santa Cruz Biotechnology, sc-2762, 1:300) https://www.scbt.com/p/normal-mouse-igg-b<br>HIF-1α (anti-rabbit, Cell Signaling Technologies, #3716, 1:1000) https://www.cellsignal.com/products/primary-antibodies/hif-1a-antibody/3716<br>HIF-2α (anti-rabbit, Abcam, ab199, 1:500) https://www.abcam.com/hif-2-alpha-antibody-ab199.html<br>EHD2 (anti-rabbit, Abcam, ab154784, 1:1000) https://www.abcam.com/ehd2-antibody-epr9821-ab154784.html<br>EHD2 (anti-rabbit, Invitrogen, PA5-49403, 1:1000) https://www.thermofisher.com/antibody/product/EHD2-Antibody-Polyclonal/PA5-49403<br>β-actin (anti-mouse, Sigma-Aldrich, A5316, 1:2500) https://www.sigmaaldrich.com/SE/en/product/sigma/a5316<br>GLUT1 (anti-rabbit, Abcam, ab15309, 1:100) https://www.abcam.com/glucose-transporter-glut1-antibody-ab15309.html<br>Na-K-ATPase (anti-rabbit, Abcam, ab76020, 1:100) https://www.abcam.com/sodium-potassium-atpase-antibody-ep1845y-plasma-membrane-loading-control-ab76020.html<br>FITC conjugated to anti-pimonidazole mouse IgG1 monoclonal antibody (FITC-MAb1) (anti-mouse, Hypoxyprobe, HP2-100Kit, 1:100) http://www.hypoxyprobe.com/hp2-100kit.html<br>Goat-anti-Rabbit IgG (H+L), Alexa Fluor488 (Invitrogen, A27034, 1:600) https://www.thermofisher.com/antibody/product/Goat-anti-Rabbit-IgG-H-L-Secondary-Antibody-Recombinant-Polyclonal/A27034<br>Goat anti-Rabbit IgG (H+L) Highly Cross-Adsorbed Secondary Antibody, Alexa Fluor Plus 647 (Invitrogen, A32733, 1:600) https://www.thermofisher.com/antibody/product/Goat-anti-Rabbit-IgG-H-L-Highly-Cross-Adsorbed-Secondary-Antibody-Polyclonal/A32733<br>CD3 antibody (anti-mouse, Biolegend, 100205, 1:100) https://www.labome.com/product/BioLegend/100205.html<br>CD45 antibody (anti-mouse, Biolegend, 103127, 1:100) https://www.biolegend.com/en-us/products/alexa-fluor-700-anti-mouse-cd45-antibody-3407?GroupID=BLG6833<br>CD4 antibody (anti-mouse, Biolegend, 100429, 1:100) https://www.biolegend.com/en-us/products/alexa-fluor-700-anti-mouse-cd4-antibody-3385?GroupID=BLG4745<br>CD8 antibody (anti-mouse, Biolegend, 126619, 1:100) https://www.biolegend.com/en-us/products/apc-cyanine7-anti-mouse-cd8b-antibody-10021<br>CD11b antibody (anti-mouse, Biolegend, 101205, 1:100) https://www.biolegend.com/en-us/products/fitc-anti-mouse-human-cd11b-antibody-347?GroupID=BLG10660<br>IAIE antibody (anti-mouse, Biolegend, 107625, 1:100) https://www.biolegend.com/en-us/search-results/percp-cyanine5-5-anti-mouse-i-a-i-e-antibody-4282<br>F4/80 antibody (anti-mouse, Biolegend, 123115, 1:100) https://www.biolegend.com/en-us/products/apc-anti-mouse-f4-80-antibody-4071?GroupID=BLG5319<br>Gr1 antibody (anti-mouse, Biolegend, 108415, 1:100) https://www.biolegend.com/en-us/products/pe-cyanine7-anti-mouse-ly-6g-ly-6c-gr-1-antibody-1931?GroupID=BLG4876<br>CD11c antibody (anti-mouse, Biolegend, 117307, 1:100) https://www.biolegend.com/en-us/products/pe-anti-mouse-cd11c-antibody-1816?GroupID=BLG11937

Eukaryotic cell lines

Policy information about cell lines

Cell line source(s)	MHCC97L cell line was a gift from Dr Z.Y. Yang of Fudan University (Li et al. 2001. World J Gastroenterol. 7(5): 630-6). PLC/PRF/5, Hep3B, MIHA and THLE3 cell lines were purchased from American Type Culture Collect (ATCC).
Authentication	All cell lines used were authenticated using the AuthentiFiler PCR Amplification Kit (Applied Biosystems) according to the manufacturer's instructions.
Mycoplasma contamination	All cell lines used were tested for mycoplasma contamination by PCR amplification of mycoplasma genetic material. Mycoplasma contamination was not found any of the cell lines
Commonly misidentified lines<br>(See ICLAC register)	None of the cell lines used are registered as commonly misidentified cell lines.

Animals and other organisms

Policy information about studies involving animals; ARRIVE guidelines recommended for reporting animal research

Laboratory animals	BALB/cAnN-nu (nude) mice, male, 6-8 weeks old<br>C57BL/6N mice/C57BL/6N, male, 8-10 weeks old<br>C57BL/6N Ehd2 transgenic KO (Ehd2-/-), male, 8-10 weeks old<br>Mice were housed with a dark/light cycle of 12 hour, ambient temperature of 22°C and humidity of 30-70%.
Wild animals	The study did not involve wild animals.
Field-collected samples	The study did not involve field-collected samples.
Ethics oversight	The study protocols were approved by were approved by the Committee on the Use of Live Animals in Teaching and Research (CULATR) of The University of Hong Kong and adhered to the Animals (Control of Experiments) Ordinance of Hong Kong.

Note that full information on the approval of the study protocol must also be provided in the manuscript.

Human research participants

Policy information about studies involving human research participants

Population characteristics	The HCC patients are Chinese with a mean age of 53.98 years (SD=11.76). There were 64 men and 20 women. The patients were diagnosed as HCC at Queen Mary Hospital, The University of Hong Kong. Tumors were staged according to the pathological tumor-nodemetastasis (pTNM) staging system.
Recruitment	Human HCC and their corresponding nontumorous liver samples were collected at the time of surgical resection at Queen Mary Hospital, The University of Hong Kong, from 1991 to 2008.
Ethics oversight	Prior approval for the use of clinical tissue samples was acquired from the Institutional Review Board of The University of Hong Kong and the Hospital Authority of Hong Kong. The patients signed consent forms to acknowledge the use of their resected tissues for research purposes.

Note that full information on the approval of the study protocol must also be provided in the manuscript.

Flow Cytometry

Plots

Confirm that:

- [x] The axis labels state the marker and fluorochrome used (e.g. CD4-FITC).
- [x] The axis scales are clearly visible. Include numbers along axes only for bottom left plot of group (a 'group' is an analysis of identical markers).
- [x] All plots are contour plots with outliers or pseudocolor plots.
- [x] A numerical value for number of cells or percentage (with statistics) is provided.

Methodology

Sample preparation	Immortalized human HCC cells were trypsinized into single cell suspensions. Mouse tumor tissues were dissociated into single cell suspensions by gentleMACSdissociator in serum-free DMEM-F12 with DNase1 and Liberase. Red blood cells were lysed with ACK lysis buffer. After treatments with Fc Block, antibodies were added to cell suspensions for staining different cell surface markers for 30 minutes at 4 degree.
Instrument	BD LSRFortessa flow cytometer was used for data collection.
Software	FlowJo v10.7 (FlowJo, LLC.) software was used to analyze flow cytometry results.
Cell population abundance	Flow cytometry was performed on single cell suspension from human HCC cells or dissociated mouse HCC tissues, at least 100,000 cells were collected per sample. Cell sorting was not performed.
Gating strategy	Cells were gated from FSC-A and SSC-A for the human HCC cells or live cells dissociated from mouse liver tumor tissues. Different immune cell panels were gated from distinct surface markers. CD4+ T-cell population was defined as CD3+CD4+/CD45+%, CD8+ T-cell population was defined as CD3+CD8+/CD45+%, macrophage population was defined as F4/80+/CD45+%, MDSC population was defined as CD11b+Gr1+/CD45+%, DC population was defined as CD11b+CD11c+/CD45+% or IAIE+/CD11c+%.

- [x] Tick this box to confirm that a figure exemplifying the gating strategy is provided in the Supplementary Information.
